# Dynamics of a neuron–glia system: the occurrence of seizures and the influence of electroconvulsive stimuli: A mathematical and numerical study

**DOI:** 10.1007/s10827-020-00746-5

**Published:** 2020-05-12

**Authors:** André H. Erhardt, Kent-Andre Mardal, Jakob E. Schreiner

**Affiliations:** 1 Department of Mathematics, University of Oslo, P.O.Box 1053 Blindern, 0316 Oslo, Norway; 2 Department of Computational Physiology, Simula Research Laboratory, 1325 Lysaker, Norway; 3 Expert Analytics AS, Tordenskiolds gate 3, 0160 Oslo, Norway

**Keywords:** Nonlinear dynamics, Bifurcation theory, Neuron–glia cell system, Monodomain and bidomain model, Seizure, Electroconvulsive therapy (ECT)

## Abstract

In this paper, we investigate the dynamics of a neuron–glia cell system and the underlying mechanism for the occurrence of seizures. For our mathematical and numerical investigation of the cell model we will use bifurcation analysis and some computational methods. It turns out that an increase of the potassium concentration in the reservoir is one trigger for seizures and is related to a torus bifurcation. In addition, we will study potassium dynamics of the model by considering a reduced version and we will show how both mechanisms are linked to each other. Moreover, the reduction of the potassium leak current will also induce seizures. Our study will show that an enhancement of the extracellular potassium concentration, which influences the Nernst potential of the potassium current, may lead to seizures. Furthermore, we will show that an external forcing term (e.g. electroshocks as unidirectional rectangular pulses also known as electroconvulsive therapy) will establish seizures similar to the unforced system with the increased extracellular potassium concentration. To this end, we describe the unidirectional rectangular pulses as an autonomous system of ordinary differential equations. These approaches will explain the appearance of seizures in the cellular model. Moreover, seizures, as they are measured by electroencephalography (EEG), spread on the macro–scale (*cm*). Therefore, we extend the cell model with a suitable homogenised monodomain model, propose a set of (numerical) experiment to complement the bifurcation analysis performed on the single–cell model. Based on these experiments, we introduce a bidomain model for a more realistic modelling of white and grey matter of the brain. Performing similar (numerical) experiment as for the monodomain model leads to a suitable comparison of both models. The individual cell model, with its seizures explained in terms of a torus bifurcation, extends directly to corresponding results in both the monodomain and bidomain models where the neural firing spreads almost synchronous through the domain as fast traveling waves, for physiologically relevant paramenters.

**Electronic supplementary material:**

The online version of this article (10.1007/s10827-020-00746-5) contains supplementary material, which is available to authorized users.

## Introduction

The aim of this paper is the mathematical and numerical analysis of a neuron–glia cell system based on the models in Barreto and Cressman (2011) and Cressman et al. (2009), consisting of a single conductance–based neuron together with intra- and extracellular ion concentration dynamics. Such conductance–based models are based on an equivalent circuit representation of a cell membrane. In general, action potentials (APs) of excitable biological cells such as neurons and cardiac muscle cells are often modelled as a system of ordinary differential equations (ODEs) using a Hodgkin–Huxley (type) formalism Hodgkin and Huxley (1952). In Cressman et al. (2009) the authors studied a reduced model to find that the competition between intrinsic neuronal currents, sodium–potassium pumps, glia and diffusion can produce very slow and large amplitude oscillations in ion concentrations similar to what is seen physiologically in seizures, cf. also Barreto and Cressman (2011). Furthermore, the main focus in Cressman et al. (2009) are the sodium and potassium dynamics of the considered system. Moreover, as mentioned in Barreto and Cressman (2011), the effects of extracellular potassium concentration [*K*]_*o*_ accumulation on neuronal excitability have long been recognised, and deficiencies in [*K*]_*o*_ regulation have been implicated in various types of epilepsy and spreading depression, e.g. in Somjen et al. (2008a). More recently, computational studies have begun to clarify the role of impaired [*K*]_*o*_ regulation, cf. Kager et al. (2007) and Somjen et al. (2008b), as well as other varying ion concentrations, e.g. in Cressman et al. (2009) and Ullah et al. (2009). Notice that for instance in Barreto and Cressman (2011) the authors are simplifying their model by the consideration of fixed ion concentrations of the extracellular potassium concentration [*K*]_*o*_ and intracellular sodium concentration [*Na*]_*i*_, while our study is focused on the dynamics of the full system and we want to avoid simplifications to derive a general analysis of the considered model.

Our main focus is threefold. First of all, we will investigate the potassium dynamics, e.g. the effect of the extracellular potassium concentration [*K*]_*o*_. It is well known that [*K*]_*o*_ has an influence on the occurrence of seizures, cf. (Fröhlich et al. 2006, 2008; Krishnan and Bazhenov 2011; Krishnan et al. 2015; Du et al. 2016; González et al. 2019). Additionally, our analysis of the cell model is not only focused on an increase of the extracellular potassium concentration to gain seizures. We will show that seizures appear in the neighbourhood of a torus bifurcation, which entails also with an increase in the potassium concentration in the reservoir and finally, also in the extracellular potassium concentration. However, we will point out that the ”standard” approach — i.e. the reduction of the dimension of the ODE system by removing one differential equation, e.g. for the extracellular potassium concentration and using the extracellular potassium concentration as bifurcation parameter — may exclude the torus bifurcation from the bifurcation diagram. The second goal is focused on the effect of an external stimulus, more specifically unidirectional rectangular pulses (Peterchev et al. 2010). Therefore, we will model a suitable unidirectional rectangular pulse and we will derive an externally excited cell model. Finally, we will use our results from the bifurcation analysis for the cell model, i.e. the ion current interaction and the effect of an external stimulus for an extension to the macro–scale (*cm*), since seizures spread on the macro–scale (*cm*). A more thorough understanding of the interaction of complex cell models (ODEs) with the spatial models (PDEs) is needed before looking at seizures on a macro–scale (*cm*). Summarising, we will derive a suitable cell model based on the models in Barreto and Cressman (2011) and Cressman et al. (2009), including unidirectional rectangular pulses, and we will analyse the model regarding the appearance of seizures. With these results in mind, we will derive a monodomain model to extrapolate the cellular behaviour to the macro–scale, i.e. a model of partial differential equations (PDEs) coupled with the ODE system and investigate whether the model is adequate to investigate seizures. The coupled ODE–PDE system is solved using cbcbeat, described in Rognes et al. (2017). Furthermore, we will extend our study from the monodomain model to a bidomain model to be able to model a more realistic structure (of parts) of the brain. The bidomain model is already extensively used in the cardiac modelling, cf. Sundnes et al. (2006), and one advantage is that it takes into account the anisotropy of both the intracellular and extracellular spaces, which potentially is important for the modelling of the brain. In addition, it allows us to model the different regions of the brain, i.e. the grey and white matter. The grey matter is a major component of the central nervous system, consisting of e.g. neuronal cell bodies and glial cells, while the white matter contains relatively very few cell bodies. Therefore, there is a significant difference between cardiac and neuronal models and additionally, the cardiac spiking has a lower frequency as the neuronal spiking. The neuronal spiking is up to 10 times faster than the cardiac one comparing normal action potentials in a single cell. Furthermore, a neuron may has also long silent periods without spiking, while the cardiac cell exhibits a continuous/periodic spiking. Moreover, the electrical potential of the heart exhibits a synchronous behaviour, while the neuronal firing in the brain is usually not synchronous. Therefore, one can except different behaviour from models of a heart or a brain. A further step towards a more realistic modelling of the brain will be done by introducing a more complex geometry for the bidomain, which mimics the shape of the grey and white matter. To what extents the cell model can be extended to macro–scale models using the monodomain or bidomain equations and to what extents these equations alters the behaviour of the cell model has not been adequately addressed yet and is hence one of the topics of this paper.

The paper is organised as follows: We will start with brief subsections on the biological background and the modelling of the considered system of ODEs in Section 1.1 and Section 1.2. In Section 1.3 we show how to model an external forcing (electroconvulsive therapy (ECT) stimuli) as unidirectional rectangular pulses (Peterchev et al. 2010), where one can model similarly bidirectional rectangular pulses. Moreover, we will show the influence of this external stimulus, which might have an effect on the system. After the introduction, we will go on with our main investigation in Section 2. For our study we will use bifurcation theory and numerical bifurcation analysis, since it provides a strategy for investigating the behaviour of the considered system of ODEs. In general, a bifurcation of a dynamical system is a qualitative change in its dynamics produced by varying parameters. A very good introduction into this topic can be found in Kuznetsov (1998), where the author does not only explain and discuss the bifurcation theory, he also provides the numerical background for the numerical bifurcation analysis, cf. also Shilnikov et al. (1998,2001). Furthermore, we will derive the suitable bifurcation diagram utilising MATLAB together with the toolboxes MATCONT and CL_MATCONT (Dhooge et al. 2003, 2008; Govaerts et al., 2005), which are numerical continuation packages for the interactive bifurcation analysis of dynamical systems. For our analysis we use MATLAB R2018a and the MATCONT version *matcont6p11*. Here, we want to highlight that numerical bifurcation analysis is already used for the investigation of neurons, see e.g. (Izhikevich 2000; Tsaneva-Atanasova et al. 2005) and (Atherton et al. 2016; Krupa et al. 2014; Rotstein et al. 2006) to mention only a few papers. The authors consider in many cases a reduced subsystem of lower dimension, cf. for a general introduction (Desroches et al. 2012; Kuehn 2015), which can be derived by a time scale separation argument, cf. Rubin and Wechselberger (2007), Rubin and Terman (2004), and Wang and Rubin (2016). These subsystems are easier to analyse and give an insight into the behaviour of the system during slow and fast epochs. A similar approach is also used for the investigations of neuron and glia models, cf. Hübel et al. (2016) and Østby et al. (2009). But this approach does not necessarily explain the complete behaviour of the system, cf. Erhardt (2019). However, our aim is to study the full system and its behaviour using numerical bifurcation analysis as in Erhardt (2018) and Tsaneva-Atanasova et al. (2010). Therefore, we will study on the one hand the full system, and on the other hand we will investigate the potassium dynamics considering a reduced system to be able to explain the mechanism behind seizures. In Section 3, we will extend our results from the cellular level to the macro–scale (*cm*). From electroencephalography it is clear that a seizure involves the interaction between cells on a macro–scale. To this end, we will consider a homogenised monodomain model extending the single cell model with a PDE describing the electrical conductance in the brain on the macro–scale. We will perform a set of numerical experiments to establish how the time–scales from the bifurcation analysis are affected by this spatial coupling. The key part of our experiments is the need for an unstable spatial region in which seizures are initiated and the time–scales involved. This instability can be for instance a region of high bath *K*_bath_ concentration, or a spatially focused external electrical stimulus. Furthermore, we will extend our study from the monodomain model to the bidomain model to derive a more realistic macro–scale model, cf. Section 3.2. Finally, in Section 4 we will discuss our results. The main novelty of our work is that we study a neuron–glia cell system without a reduction of the dimension of the system, except if we study the potassium dynamics using [*K*]_*o*_ as bifurcation parameter to compare the different bifurcation diagrams to show that this approach does not explain all details. Moreover, we do not use any further simplifications and we additionally extend the system to an external forced system. A last novelty is that we develop, based on our cellular model in combination with our analysis, two macro–scale models describing seizures on the tissue level which enable the prediction of time–scales and propagation velocities of seizure spreading on the macro–scale.

### Mathematical models of the electrophysiology

In this section we give a brief introduction to the biological background, the mathematical modelling and the treatment of an external stimulus. Basically, the intrinsic excitability of neuronal networks depends on the reversal potentials for various ion species as it is mentioned in Cressman et al. (2009). The reversal potentials in turn depend on the intra- and extracellular concentrations of the corresponding ions. During neuronal activity, the extracellular potassium concentration [*K*]_*o*_ and intracellular sodium concentration [*Na*]_*i*_ increase. Glia help to reestablish the normal ion concentrations. Consequently, neuronal excitability is transiently modulated in a competing fashion: the local increase in [*K*]_*o*_ raises the potassium reversal potential, increasing excitability, while the increase in [*Na*]_*i*_ leads to a lower sodium reversal potential and thus less ability to drive sodium into the cell. The relatively small extracellular space and weak sodium conductances at normal resting potential can cause the transient changes in [*K*]_*o*_ to have a greater effect over neuronal behaviour than the changes in [*Na*]_*i*_, and the overall increase in excitability can cause spontaneous neuronal activity. For more details we refer to Cressman et al. (2009). The full model in Cressman et al. (2009) consists of one single–compartment conductance–based neuron containing a sodium, potassium, calcium–gated potassium, and leak currents, augmented with dynamic variables representing the intracellular sodium and extracellular potassium concentrations. This mechanism can be mathematically modelled as described in Section 1.2. Such a conductance–based model is based on an equivalent circuit representation of a cell membrane and represents the action potential of a neuron, which is a temporary, characteristic variance of the membrane potential from its resting potential. The molecular mechanism of APs is based on the interaction of voltage–sensitive ion channels. The reason for the formation and special properties of APs is established in the properties of different groups of ion channels in the plasma membrane. The electrophysiological behaviour of an excitable biological cell can be described with the following differential equation/system

$$ C_{m}\frac{\mathrm{d} V}{\mathrm{d}t}=-I_{\text{ion}}+I_{\text{stimulus}}, $$

where *V* denotes the voltage (in *mV*) and *t* the time (in *ms*), *C*_*m*_ is the membrane capacitance, and *I*_ion_ is the sum of all the membrane currents and the external stimulus respectively. The different ion currents may depend on gating variables, which are important for the opening (activation) and closing (inactivation) of the different ion currents, see Section 1.2 for more details.

Furthermore, the aim of this manuscript is the extension of our results on the cellular level to the macro–scale level. To achieve this goal we will also study the corresponding monodomain model, i.e.

$$ C_{m}\frac{\partial V}{\partial t}=-I_{\text{ion}}+\frac{\lambda}{1+\lambda}\frac{1}{\chi}\nabla\cdot\left( M_{i}\nabla V\right), $$

where *M*_*i*_ denotes the intracellular conductivity tensor, *λ* the extra- to intracellular conductivity ratio and *χ* the membrane surface area per unit volume. On the macro–scale (*cm*) we assume that all neurons behaves similar and are connected through a diffusion process involving the transmembrane potential, thus we are considering a homogenised model. Notice that without the spatial dependency the monodomain model again is reduced to the ODE model describing the cellular mechanism. For more details, see e.g. Keener and Sneyd (1998) and Sundnes et al. (2006) and Section 3. With this model it is interesting to quantify the velocity and the extent to which a seizure spreads from an unstable region into a stable region. Moreover, the model can also decide whether the time–scales involved in the seizures at the cellular level is affected by the neighbouring cells under physiologically reasonable conditions. Macro–scale simulations, even based on patient–specific geometries have been performed, but to the authors’ knowledge the only attempt at studying seizures is through modelling the brain as a passive conductor, linking the magnitude of the electrical potential to the initiation of seizures, see Lee et al. (2016). In Dougherty et al. (2014) the authors used the bidomain model – typically used in cardiac modelling, see Sundnes et al. (2006) – to study neural activation, but without looking at seizures. Finally, we will extend our study also to the following bidomain model to be able to capture more features during a seizure on macro–scale:

$$ C_{m} \frac{\partial V}{\partial t}= -I_{\text{ion}}+\frac{1}{\chi}(\nabla \cdot (M_{i} \nabla V)+\nabla \cdot (M_{i} \nabla U_{e}) ) $$

with

$$ \nabla\cdot(M_{i}V)+\nabla((M_{i}+M_{e})\nabla U_{e})=0, $$

where *M*_*i*_ and *M*_*e*_ denote the intracellular and extracellular conductivity tensor, respectively, while *U*_*e*_ is the extracellular potential. At this stage, one can see that the bidomain model takes into account the anisotropy of both the intracellular and extracellular spaces. Furthermore, both models are equipped with suitable Neumann boundary conditions, which also reflect the different anisotropies, cf. Section 3.

### The mathematical model

Here, we state a mathematical model based on the models in Barreto and Cressman (2011); Cressman et al.(2009, 2011), which we will use for our investigations. As already mentioned the system includes the sodium current

$$ I_{\text{Na}} = \left( G_{\text{NaL}}+G_{\text{Na}}m^{3}h\right)(V-E_{\text{Na}}), $$

the chloride current *I*_Cl_ = *G*_ClL_(*V* − *E*_Cl_) and the potassium current

$$ I_{\text{K}} = \left( G_{\text{K}}n^{4}+G_{\text{AHP}}\frac{[Ca]_{i}}{1+[Ca]_{i}}+G_{\text{KL}}\right)(V-E_{\text{K}}), $$

where the different conductances are stated in Table 1. Moreover, the capacity of glial cells to remove excess potassium from the extracellular space is modelled by

$$ I_{\text{glia}}=\left( \frac{G_{\text{glia}}}{1+\exp\left( \frac{18-[K]_{o}}{2.5}\right)}\right), $$

while the potassium diffusion to the nearby reservoir is represented by the current *I*_diff_ = *ε*([*K*]_*o*_ − *K*_bath_), *ε* = 1.2 *Hz* and *K*_bath_ = 4 *mM*, where *K*_bath_ denotes the potassium concentration in the reservoir. Furthermore, the membrane potential *V* has unit *mV*, while the time *t* has unit *ms* and the different ion concentrations are given in *mM*. The full model reads as follows:

1 $$ \begin{array}{@{}rcl@{}} C_{m}\frac{\mathrm{d} V}{\mathrm{d}t} &=& -(I_{\text{Cl}}+I_{\text{Na}}+I_{\text{K}}),\\ \frac{\mathrm{d} y}{\mathrm{d}t}&=& 3\frac{y_{\infty}-y}{\tau_{y}},\\ \frac{\mathrm{d} [Ca]_{i}}{\mathrm{d}t}&=&-\frac{[Ca]_{i}}{80}-G_{\text{Ca}}\frac{0.002(V-E_{\text{Ca}})}{1+\exp\left( -\frac{25+V}{2.5}\right)},\\ \frac{\mathrm{d} [K]_{o}}{\mathrm{d}t}&=& -\frac{1}{\tau}(I_{\text{diff}}+14 I_{\text{pump}}+I_{\text{glia}}-7\gamma I_{\text{K}}),\\ \frac{\mathrm{d} [Na]_{i}}{\mathrm{d}t}&=& -\frac{1}{\tau}\left( \gamma I_{\text{Na}}+3I_{\text{pump}}\right), \end{array} $$

where *y* represents the gating variables *h*, *m* and *n*, $C_{m}=1\ \frac {\mu F}{cm^{2}}$ is the membrane capacity, *τ* = 1000 is used to convert *s* into *ms*, *γ* = 0.0445 is a unit conversion factor that converts the membrane currents into $\frac {mM}{s}$. The expression *I*_pump_ denotes the sodium–potassium pump given by

$$ I_{\text{pump}}=\frac{\varrho}{1+\exp\left( 5.5-[K]_{o}\right)}\left( \frac{1}{1+\exp\left( \frac{25-[Na]_{i}}{3}\right)}\right) $$

with $\varrho = 1.25\ \frac {mM}{s}$, while the time relaxation constant of the corresponding gating variable and its steady state are denoted by

$$ \tau_{y}(V)=\frac{1}{a_{y}(V)+b_{y}(V)} \ \text{and} \ y_{\infty}(V)=\frac{a_{y}(V)}{a_{y}(V)+b_{y}(V)}, $$

respectively, where the functions *a*_*y*_ and *b*_*y*_ for the gating variables are given by

$$\begin{array}{ll} a_{m}=\frac{0.1(V+30)}{1-\exp(-\frac{V+30}{10})}, \ &b_{m}=4\exp\left( -\frac{V+55}{18}\right), \\ a_{h}=0.07\exp\left( -\frac{V+44}{20}\right), \ &b_{h}=\frac{1}{1-\exp(-\frac{V+14}{10})}, \\ a_{n}=\frac{0.01(V+34)}{1-\exp(-\frac{V+34}{10})}, \ &b_{n}=\frac{1}{8}\exp\left( -\frac{V+44}{80}\right). \end{array} $$

Moreover, the Nernst potentials of the ion currents are given by

$$ E_{\text{Na}}=26.64\log\left( \frac{[Na]_{o}}{[Na]_{i}}\right), \ E_{\text{K}}=26.64\log\left( \frac{[K]_{o}}{[K]_{i}}\right), $$

$$ E_{\text{Cl}}=26.64\log\left( \frac{[Cl]_{i}}{[Cl]_{o}}\right), $$

*E*_Ca_ = 120 *mV* with [*Cl*]_*i*_ = 6 *mM*, [*Cl*]_*o*_ = 130 *mM*, [*K*]_*i*_ = 158 *mM* − [*Na*]_*i*_ and [*Na*]_*o*_ = 270 *mM* − 7[*Na*]_*i*_, cf. Eqs. (2). Notice that system (1) is of dimension 7, while the models from Barreto and Cressman (2011) and Cressman et al. (2009) are 5 or 6 dimensional systems.

**Table 1:** Further system parameters (default setting)

*G*_glia_	*G*_Na_	*G*_NaL_	*G*_K_	*G*_KL_	*G*_ClL_	*G*_Ca_	*G*_AHP_
$66 \frac {mM}{s}$	$100 \frac {mS}{cm^{2}}$	$0.0175 \frac {mS}{cm^{2}}$	$40 \frac {mS}{cm^{2}}$	$0.05 \frac {mS}{cm^{2}}$	$0.05 \frac {mS}{cm^{2}}$	$0.1 \frac {mS}{cm^{2}}$	$0.01 \frac {mS}{cm^{2}}$

These parameters we will always use for our simulations unless we specify a new setting for different parameters

**Fig. 1:**
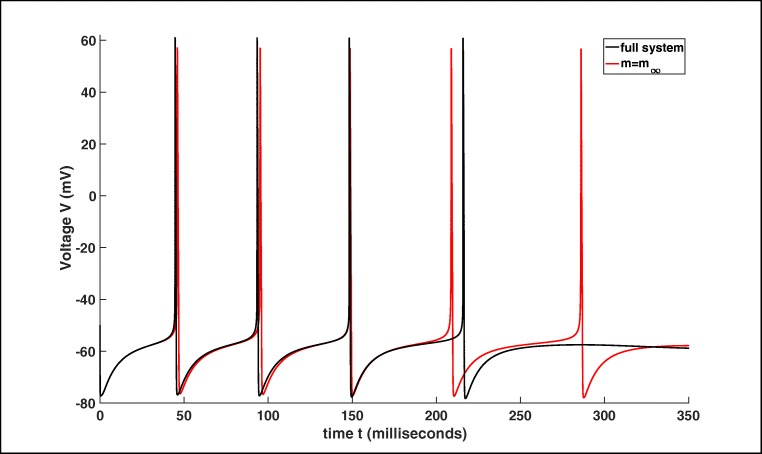
Comparison of system (1) (full system) with the simplified model using $m=m_{\infty }$. In both cases we are utilising the same parameters and initial values, see Tables 1 and 2

**Fig. 2:**
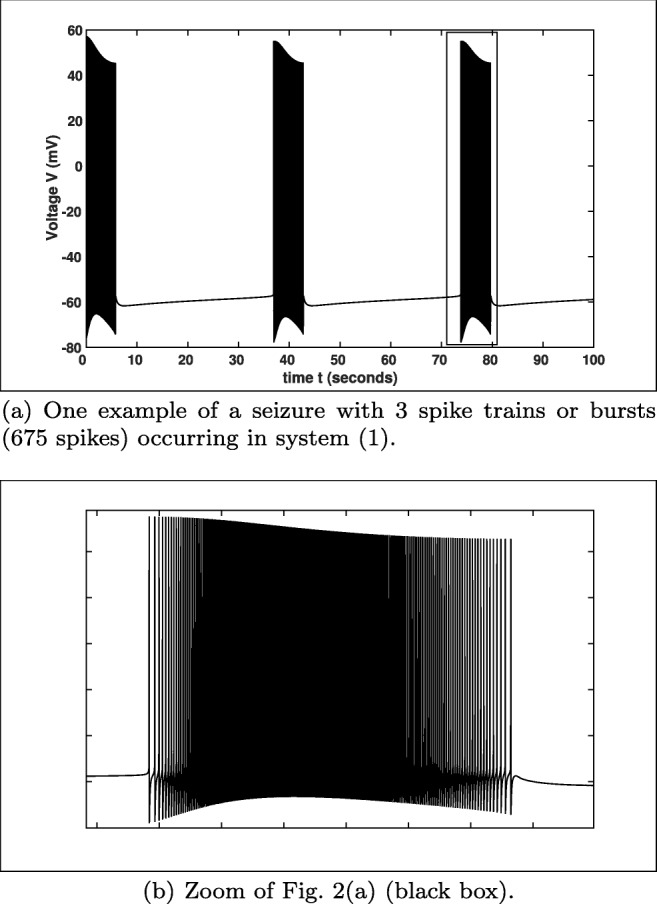
Simulation of system (1) with *K*_bath_ = 8 *mM* showing a seizure.

In Barreto and Cressman (2011) and Cressman et al. (2009) the authors assume that the gating variable *m* is equal to its steady state $m_{\infty }$, while we consider *m* as additional state variable and thus, the dimension of our system is at least one dimension higher as the one in Barreto and Cressman (2011) and Cressman et al. (2009). The intracellular potassium, extracellular sodium and chloride concentrations are obtained in Cressman et al. (2009) by the following expressions:

2 $$ \begin{array}{@{}rcl@{}} {[}K{]}_{i}&=&152\ mM-([Na]_{i}-[Cl]_{i}),\\ {[}Na{]}_{o}&=&144\ mM-\beta([Na]_{i}-18\ mM),\\ {[}Cl{]}_{o}&=&130\ mM-\beta([Cl]_{i}-6\ mM), \end{array} $$

where *β* = 7 is the ratio of the intracellular to extracellular volume, cf. also Wei et al. (2014a) and Wei et al. (2014b). For more details we refer to Barreto and Cressman (2011) and Cressman et al. (2009). The different APs of the full system (1) and the reduced version with $m=m_{\infty }$, we compare in Fig. 1. Here, we see that the general behaviour is similar using the initial values from Table 2, but the trajectory of both systems have a slightly different period and the number of APs (spikes) is different. Furthermore, at the default parameter values from Table 1 both systems approach a stable resting state for which the membrane voltage and the ion concentrations assume fixed values, cf. Barreto and Cressman (2011). This behaviour changes for instance by increasing the value of *K*_bath_, see one example in Fig. 2. Figure 2 shows an example for a seizure (i.e. 3 spike trains or bursts with 675 spikes in 100 *ms*) which was expected for *K*_bath_ = 8 *mM* as stated in Wei et al. (2014b).

**Table 2:** Initial values (default setting)

*V*_init_	*m*_init_	*h*_init_	*n*_init_	[*Ca*]_*i*__init_	[*K*]_*o*__init_	[*Na*]_*i*__init_
− 50 *mV*	0.0936	0.96859	0.08553	0.0 *mM*	7.8 *mM*	15.5 *mM*

These initial values we will always utilise for our simulations unless we specify them differently

Furthermore, regarding system (1) we see that our model is dependent on several parameters which might have a big influence on the occurrence of seizures and the general behaviour of the system. This behaviour we will analyse and investigate systematically in Section 2 using (numerical) bifurcation analysis.

**Fig. 3:**
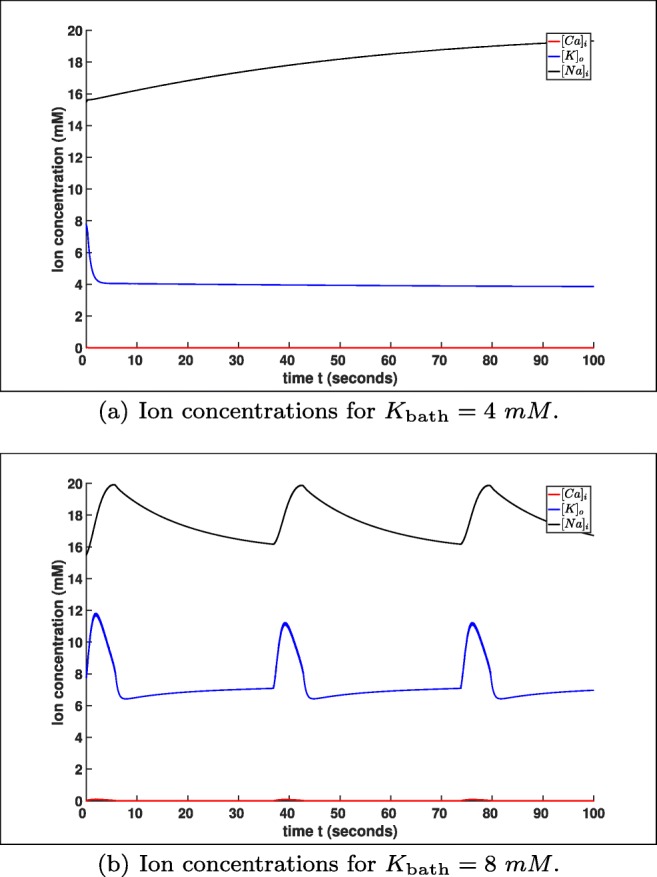
Comparison of the ion concentrations of model (1) for *K*_bath_ = 4 *mM* and *K*_bath_ = 8 *mM*

Furthermore, the ion concentrations [*Ca*]_*i*_, [*K*]_*o*_ and [*Na*]_*i*_ are also showing a different behaviour for *K*_bath_ = 4 *mM* (default setting) and *K*_bath_ = 8 *mM*, cf. Fig. 3. In Fig. 3 we see that the different ion concentrations have on the one hand a change in their time–scales and on the other hand also a change in their concentration variation. In Fig. 3a we see that the range of concentration variations for [*Ca*_*i*_], [*K*]_*o*_ and [*Na*]_*i*_ are very small compared to Fig. 3b. Moreover, in Fig. 3a the concentrations tend to an equilibrium similar to the membrane voltage *V* in Fig. 1, while the concentrations for *K*_bath_ = 8 *mM* are oscillating for a much longer period and a much larger amplitude, cf. Fig. 3b.

**Fig. 4:**
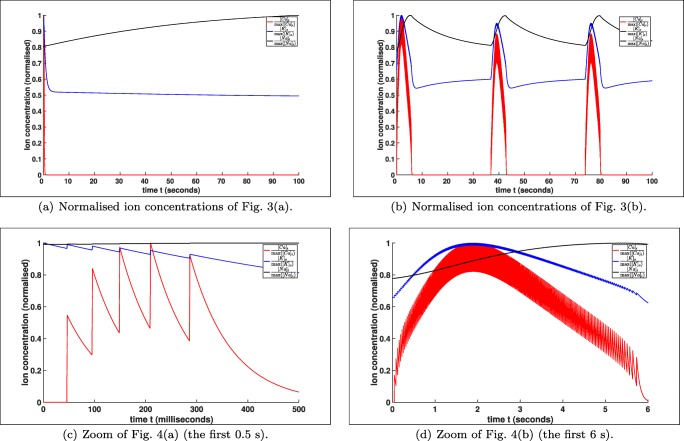
Comparison of the normalised ion concentrations of Fig. 3

This affects of course the membrane potential *V*, which has again influence on the different ion concentrations. Regarding Eqs. (2) also the ion concentrations [*K*]_*i*_ and [*Na*]_*o*_ are affected by this. For a better understanding we also compare the normalised ion concentrations in Fig. 4. In Fig. 4(a) we see also that the ion concentrations are reaching after a short firing of approximately 0.5 *s*, cf. Fig. 4c, more or less a stable resting state, while for *K*_bath_ = 8 *mM* the periodic behaviour goes on, cf. Fig. 4b and d.

### Modelling of electroconvulsive therapy (ECT) stimulus waveforms

The aim of this section is the implementation of a periodic external forcing (unidirectional rectangular pulses) of system (1), which might cause seizures, cf. Peterchev et al. (2010). To achieve this goal we have to introduce an external forcing term *I*_ext_, i.e. we will consider the following differential equation:

3 $$ C_{m}\frac{\mathrm{d} V}{\mathrm{d}t} = -(I_{\text{Cl}}+I_{\text{Na}}+I_{\text{K}})+I_{\text{ext}}. $$

First of all, we will explain the modelling of this periodic forcing term with an easy example and then, we will use this idea for our main aim. As a first remark we want to highlight that a generic non-autonomous first order ODE system which is given by

4 $$ \frac{\mathrm{d}x}{\mathrm{d}t}=f(x,t), $$

where $x=(x_{1},\dots ,x_{n})\in \mathbb {R}^{n}$ and $f:\mathbb {R}^{n+1}\to \mathbb {R}^{n}$, *n* ≥ 2, can be rewritten as an autonomous system by introducing a new variable $s\in \mathbb {R}$ as follows:

5 $$ \frac{\mathrm{d}x}{\mathrm{d}t}=f(x,s),~~~ \frac{\mathrm{d}s}{\mathrm{d}t}=1.  $$

At this stage, we see that the stability and bifurcation analysis of system (5) fail, since the system exhibits no equilibrium. In our situation we need a periodic time–dependent forcing term *I*_ext_ in Eq. (3). The simple example we have in mind is a one dimensional non–autonomous ODE:

6 $$ \frac{\mathrm{d}x}{\mathrm{d}t}=f(x)+g(t) $$

with $x\in \mathbb {R}$ and $f,g:\mathbb {R}\to \mathbb {R}$, where *g*(*t*) denotes a periodic forcing term, i.e. *g*(*t*) − *g*(*t* + *T*) = 0 with the period *T* > 0. Well known periodic functions (e.g. sinusoidal forcing) are $g(t)=\cos \limits (\omega t)$ and $g(t)=\sin \limits (\omega t)$ with a period *T* > 0, which is equal to $\frac {2\pi }{\omega }$, and a frequency $\nu =\frac {1}{T}$. Moreover, we can rewrite Eq. (6) with a periodic forcing $g(t)=A\cos \limits (\omega t-\varphi )$, where *A* > 0 denotes the amplitude and *φ* is a phase change, into an autonomous system using the following observations. First of all, we consider the system of differential equations:

7 $$ \left\{\begin{array}{ll} \frac{\mathrm{d}u}{\mathrm{d}t}&=u(1-u^{2}-w^{2})-\omega w,\\ \frac{\mathrm{d}w}{\mathrm{d}t}&=w(1-u^{2}-w^{2})+\omega u. \end{array}\right.    $$

**Fig. 5:**
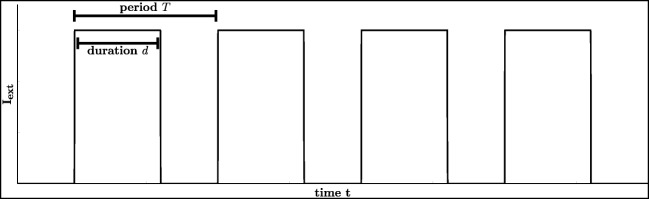
Simulation of *I*_ext_ (unidirectional pulse train): *d* = 0.6 *s*, *T* = 1 *s* and $A=3\frac {mA}{cm^{2}}$

**Fig. 6:**
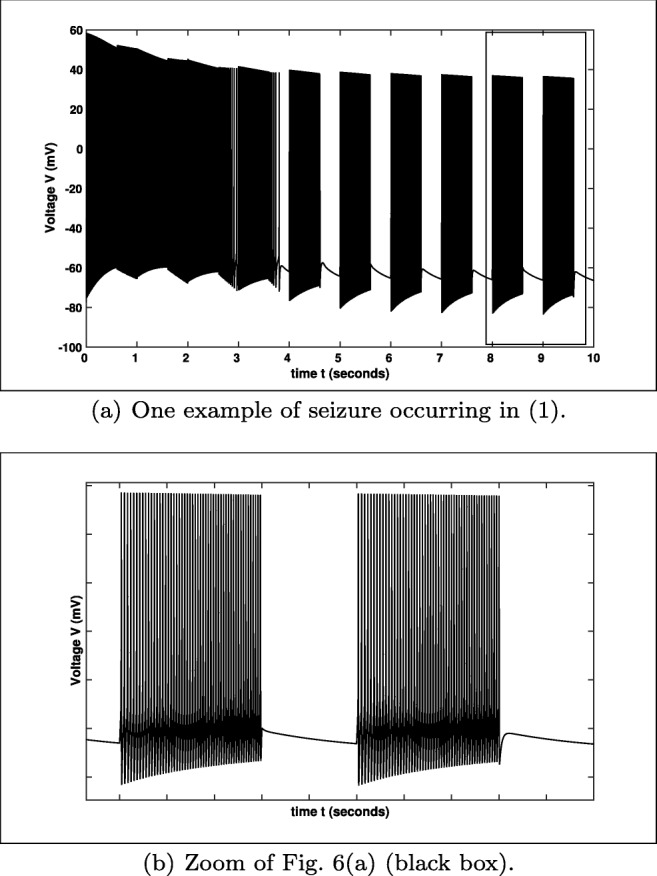
Simulation of (1) (default setting) extended with *I*_ext_ (8), cf. Fig. 5

System (7) has the solution

$$(u,w)=(\cos(\omega t+\varphi),\sin(\omega t+\varphi))$$

for any phase change *φ*. For initial values *u*(0) = 1 and *w*(0) = 0 the solution of system (7) is $(u,w)=(\cos \limits (\omega t),\sin \limits (\omega t))$. Using this we can rewrite Eq. (6) with a periodic forcing term $g(t)=A\cos \limits (\omega t-\varphi )$ as the following autonomous system:

$$ \frac{\mathrm{d}x}{\mathrm{d}t}=f(x)+A\cos(\varphi)u+A\sin(\varphi)w, $$

where *u* and *w* are the solutions of system (7) with initial values $x(0)=x_{0}\in \mathbb {R}$, *u*(0) = 1 and *w*(0) = 0 and we utilised the equality $\cos \limits (\omega t-\varphi )=\cos \limits (\varphi )\cos \limits (\omega t)+\sin \limits (\varphi )\sin \limits (\omega t)$. Our next aim is to rewrite *I*_ext_ of Eq. (3) as an autonomous differential system to arrive at an autonomous version of (3). To this end, we assume that *I*_ext_ is a periodic forcing function with frequency of 1 *Hz* and period *T* = 1000 *ms*, a suitable amplitude *A* and duration *d*. Therefore, we choose

$$ I_{\text{ext}}=\frac{A}{1+\exp(10^{2}\cdot(\cos(\varphi)-\cos(\omega t-\varphi)))} $$

with a phase change $\varphi =\frac {d\pi }{T}$ and using the previous discussion in combination with Eq. (7), we derive at

8 $$ I_{\text{ext}}=\frac{A}{1+\exp(10^{2}((1-u)\cdot\cos(\varphi)-w\cdot \sin(\varphi)))}, $$

where *u* and *w* denote the solution of Eq. (7) with initial value (*u*(0),*w*(0)) = (1,0). Please notice that the stimulus *I*_ext_ is zero for almost every time, except for *t* ∈{*k* ⋅ *T*;*k* ⋅ *T* + *d*} with *k* = 0,1,2,..., since the expression $e^{10^{2}\cdot (\cos \limits (\varphi )-\cos \limits (\omega t-\varphi ))}$ tends immediately to infinity for all *t*∉{*k* ⋅ *T*;*k* ⋅ *T* + *d*}. This is the reason for the choice in Eq. (8). Choosing *d* = 600 *ms* and $A=3\frac {mA}{cm^{2}}$ we get the graph for *I*_ext_ shown in Fig. 5. This external periodic forcing changes the behaviour as stated in Fig. 6.

Here, we see that the external forcing might have obviously an influence on the behaviour of the system – in this setting the occurrence of seizures. Notice that one can control the frequency and the duration of the occurrence of the oscillatory pattern with suitable choices of the period *T* and the duration *d*, cf. Fig. 6. Furthermore, we see that system (7) exhibits an equilibrium $(u_{0},w_{0})=(0,0)$, which influences *I*_ext_, i.e.

$$ I_{\text{ext}_{0}}=\frac{A}{1+\exp(10^{2}\cos(\varphi))}, $$

where $\varphi = \frac {d\pi }{T}$ and $\cos \limits (\varphi )>0$ for $0<\frac {d}{T}<\frac {1}{2}$, while $\cos \limits (\varphi )<0$ for $\frac {1}{2}<\frac {d}{T}<1$. Hence, $I_{\text {ext}_{0}}\approx 0$ for $0<\frac {d}{T}<\frac {1}{2}$, while $I_{\text {ext}_{0}}\approx \frac {A}{2}$ for $\frac {1}{2}<\frac {d}{T}<1$. This we will also study and analyse in the next section using the bifurcation theory together with the investigation of the influence of the different system parameters. Finally, we want to remark that we can use this approach also to model further ECT stimulus waveforms as sine wave and bidirectional rectangular pluses, cf. Peterchev et al. (2010).

## Bifurcation analysis

In the following, we will study the dynamics of system (1) regarding different system parameters. We will start with the dynamics of system (1) with respect to *K*_bath_. Moreover, we are interested in other system parameters, e.g. *G*_KL_ and we will study the influence of the external forcing *I*_ext_ (8) on the trajectory of system (1) to have a better understanding of the complex dynamics of (1). To this end we use bifurcation analysis.

First, we are going to explain our approach and then, we are investigating the specific cases. Notice that system (1) is a nonlinear system and therefore, it is difficult or impossible to derive an explicit expression of the equilibria of the system. It is quite easy to calculate the equilibria of the gating variables *h*, *m* and *n* and for the intracellular calcium concentration [*Ca*]_*i*_, i.e.

$$ h\equiv h_{\infty}(V), \ m\equiv m_{\infty}(V), \ n\equiv n_{\infty}(V) $$

and

$$ {[Ca]_{i}}_{\infty} \equiv -G_{\text{Ca}}\frac{0.16(V-E_{\text{Ca}})}{1+\exp\left( -\frac{25+V}{2.5}\right)}. $$

For the intracellular sodium concentration [*Na*]_*i*_ it is still possible to determine the equilibrium but it yields a horrible expression. At least for the membrane voltage *V* and the extracellular potassium concentration [*K*]_*o*_ one gets only an implicit term, i.e. the equilibrium of the membrane voltage *V* is determined by

$$ \begin{array}{@{}rcl@{}} &&\left( G_{\text{NaL}}+G_{\text{Na}}m_{\infty}^{3}h_{\infty}\right)(V-E_{\text{Na}})+G_{\text{ClL}}(V-E_{\text{Cl}})\\ &&+\left( G_{\text{K}}n_{\infty}^{4}+G_{\text{AHP}}\frac{{[Ca]_{i}}_{\infty}}{1+{[Ca]_{i}}_{\infty}}+G_{\text{KL}}\right)(V-E_{\text{K}})=0. \end{array} $$

Therefore, we need a numerical approach to determine the equilibria of the system, mainly as we will consider different values of *K*_bath_. Furthermore, the Jacobian $\mathcal {J}$ of the right hand side of system (1) evaluated at the equilibrium is given by

$$ \begin{array}{@{}rcl@{}} \mathcal{J} = \left( \begin{array}{lllllll} \frac{\partial F}{\partial V}&\frac{\partial F}{\partial m}&\frac{\partial F}{\partial h}&\frac{\partial F}{\partial n}&\frac{\partial F}{\partial [Ca]_{i}}&\frac{\partial F}{\partial [K]_{o}}&\frac{\partial F}{\partial [Na]_{i}}\\ \frac{1}{\tau_{m}}\frac{\partial m_{\infty}}{\partial V}&-\frac{1}{\tau_{m}}&0&0&0&0&0\\ \frac{1}{\tau_{h}}\frac{\partial h_{\infty}}{\partial V}&0&-\frac{1}{\tau_{h}}&0&0&0&0\\ \frac{1}{\tau_{n}}\frac{\partial n_{\infty}}{\partial V}&0&0&-\frac{1}{\tau_{n}}&0&0&0\\ \frac{H}{\partial V}&0&0&0&-\frac{1}{80}&0&0\\ -\frac{7\gamma}{\tau}\frac{\partial I_{\text{K}}}{\partial V}&0&0&-\frac{7\gamma}{\tau}\frac{\partial I_{\text{K}}}{\partial n}&-\frac{7\gamma}{\tau}\frac{\partial I_{\text{K}}}{\partial [Ca]_{i}}&\frac{\partial G}{\partial [K]_{o}}&\frac{\partial G}{\partial [Na]_{i}} \\ -\frac{\gamma}{\tau}\frac{\partial I_{\text{Na}}}{\partial V}&-\frac{\gamma}{\tau}\frac{\partial I_{\text{Na}}}{\partial m}&-\frac{\gamma}{\tau}\frac{\partial I_{\text{Na}}}{\partial h}&0&0&\frac{\partial L}{\partial [K]_{o}}&\frac{\partial L}{\partial [Na]_{i}} \end{array}\right), \end{array} $$

where *F* := −(*I*_Cl_ + *I*_Na_ + *I*_K_)/*C*_*m*_, *G* := −(*I*_diff_ + 14*I*_pump_ + *I*_glia_ − 7*γI*_K_)/*τ*,

$$H:=-\frac{[Ca]_{i}}{80}-G_{\text{Ca}}\frac{0.002(V-E_{\text{Ca}})}{1+\exp\left( -\frac{25+V}{2.5}\right)},$$

$L:= -\left (\gamma I_{\text {Na}}+3I_{\text {pump}}\right )/\tau $ and we used the fact that

$$ \frac{\partial}{\partial V}\left( \frac{y_{\infty}-y}{\tau_{y}}\right)=\frac{\frac{\partial y_{\infty}}{\partial V}\tau_{y}-(y_{\infty}-y)\frac{\partial \tau_{y}}{\partial V}}{{\tau_{y}^{2}}}=\frac{1}{\tau_{y}}\frac{\partial y_{\infty}}{\partial V}, $$

provided $y\equiv y_{\infty }$. Notice that varying of *K*_bath_ directly affects the extracellular potassium concentration [*K*]_*o*_, which influences *E*_K_ and therefore, indirectly the membrane voltage *V*. Finally, this affects the complete system. Moreover, varying of *K*_bath_ has also an influence on the stability of the system, which is obviously regarding the Jacobian $\mathcal {J}$. Therefore, it is of interest to study systematically the behaviour of system (1) with respect to different system parameter values, e.g. for different values of *K*_bath_. Furthermore, varying other system parameters, e.g. *G*_KL_ might have also an influence on the behaviour of the system. Some system parameters will have a big influence, e.g. *K*_bath_, and other parameters may have less influence. Furthermore, combinations of different setting will also yield different behaviours. Therefore, it is quite difficult and challenging to investigate all facets of the system behaviour. The bifurcation theory provides a very systematic approach to study the occurrence of seizures. Finally, notice that when we investigate, e.g. the potassium dynamics, i.e. we remove the corresponding ODE for [*K*]_*o*_, also the Jacobian will be reduced by the corresponding row and column, and we will use [*K*]_*o*_ as bifurcation parameter.

### Bifurcation analysis with respect to the potassium concentration in the reservoir

**Fig. 7:**
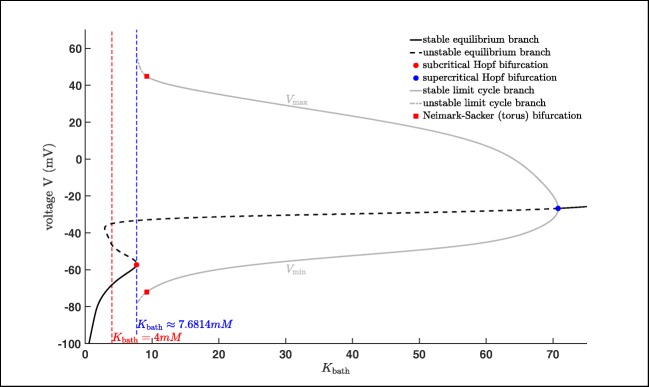
Bifurcation diagram (projection onto the (*K*_bath_,*V* )-plane) with *K*_bath_ as bifurcation parameter

Our first aim is a complete (numerical) bifurcation analysis of system (1) with respect to the potassium concentration in the reservoir *K*_bath_. The resulting bifurcation diagram will explain the behaviour of the model (1) regarding a deficit or an enhancement in *K*_bath_ and the possible occurrence of seizures. To this end, we choose *K*_bath_ as bifurcation parameter and we are determining the equilibrium curve and its stability of system (1) using the continuation algorithm from (Dhooge et al. 2003, 2008; Govaerts et al. 2005). This yields the bifurcation diagram in Fig. 7, i.e. its projection onto the (*K*_bath_,*V* )-plane. The black line shows the equilibrium curve, which is divided into two stable parts (black solid line) and one unstable part (black dashed line). Moreover, it exhibits two Andronov–Hopf bifurcation, one subcritical (red dot) and one supercritical (blue dot).

**Fig. 8:**
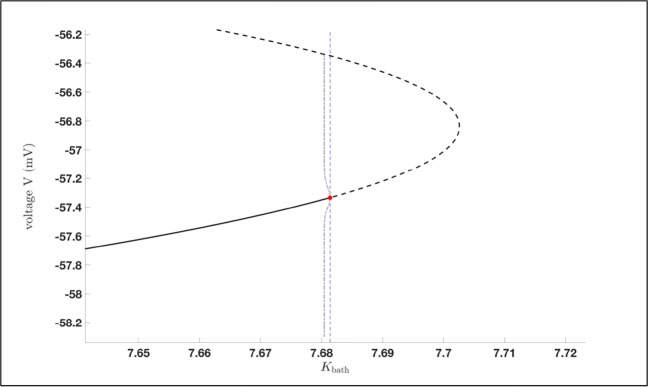
Zoom of Fig. 7 around the subcritical Andronov–Hopf bifurcation

From the supercritical Andronov–Hopf bifurcation at *K*_bath_ ≈ 70.7524 *mM* a stable limit cycle branch bifurcates, which becomes unstable via a Neimark–Sacker bifurcation or torus bifurcation at *K*_bath_ ≈ 9.2027 *mM* before it disappears at *K*_bath_ ≈ 7.6814 *mM*. From the subcritical Andronov–Hopf bifurcation at *K*_bath_ ≈ 7.6814 *mM* there is an unstable limit cycle branch bifurcating which terminates at a homoclinic bifurcation, cf. Fig. 8. A Neimark–Sacker or torus bifurcation generically corresponds to a bifurcation of an invariant torus, on which the flow contains periodic or quasi-periodic motion, cf. Ju et al. (2018). In addition, we included in Fig. 7 a red dashed line to indicate the default *K*_bath_ value which is equal to 4 *mM*. For this value we know from Fig. 1 that system (1) approaches a stable resting state. This happens for all *K*_bath_ ∈ [0;7.6814) (depending on the initial values) since only for values of *K*_bath_ between the subcritical and supercritical Andronov–Hopf bifurcation we have no stable equilibria. Furthermore, after the system loses stability the system shows several pattern of oscillations, e.g. seizures. Here, we want to highlight that varying *K*_bath_ has only a direct influence on *I*_diff_ and therefore, on the extracellular potassium concentration [*K*]_*o*_.

This obviously affects indirectly also *I*_pump_ and *I*_K_ and thus, the voltage *V* and the intracellular sodium ion concentration [*Na*]_*i*_, cf. system (1). Therefore, we will see that varying *K*_bath_ will also fit in certain ranges the potassium dynamics we investigate in Section 2.2. As we already mentioned system (1) shows seizures for increased values of *K*_bath_.

**Fig. 9:**
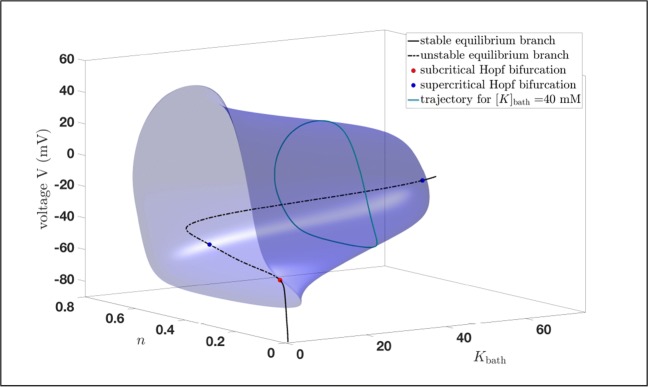
3D bifurcation diagram: Fig. 7 projection onto the (*K*_bath_,*n*,*V* )-space

More precisely, for *K*_bath_ values greater than the value of the subcritical Andronov–Hopf bifurcation and in the neighbourhood of the Neimark–Sacker or torus bifurcation yield seizures. Depending on the initial value the first firing will have a slightly different pattern, e.g. smaller or bigger amplitudes, before it converges into a stable periodic pattern as in Fig. 2 or Fig. 6. For *K*_bath_ values not close enough to the Neimark–Sacker or torus bifurcation and smaller than the value of the supercritical Andronov–Hopf bifurcation the system shows different oscillatory pattern. Here, the trajectory will converge to the stable limit cycle corresponding to the *K*_bath_ value after certain amount of time depending on the initial values, cf. Fig. 9. For instance the trajectory turns into a wave of death Wei et al. (2014b) for the *K*_bath_ value very close to the first supercritical Andronov–Hopf bifurcation. For comparison reason we will study the potassium dynamics of system (1), which is a very common approach. Nevertheless, our approach will be more general than in previous works, since we do not assume that $m\equiv m_{\infty }$. Furthermore, we will point out the relation between the potassium dynamics and the influence of the potassium concentration in the reservoir *K*_bath_.

### Potassium dynamics

After the investigation of the *K*_bath_ dynamics we will go on studying the potassium dynamics of system (1), i.e. we will consider a reduced version of this system by removing the differential equation of the extracellular potassium ion concentration [*K*]_*o*_ and then, using [*K*]_*o*_ as bifurcation parameter. Notice that all other system parameters are in the default setting. Since only *I*_diff_ is dependent on *K*_bath_ and we removed the differential equation, which is dependent on *I*_diff_, the reduced system is independent of *K*_bath_. Nevertheless, we will see that varying *K*_bath_ in the full system (1) correlates with the potassium dynamics.

**Fig. 10:**
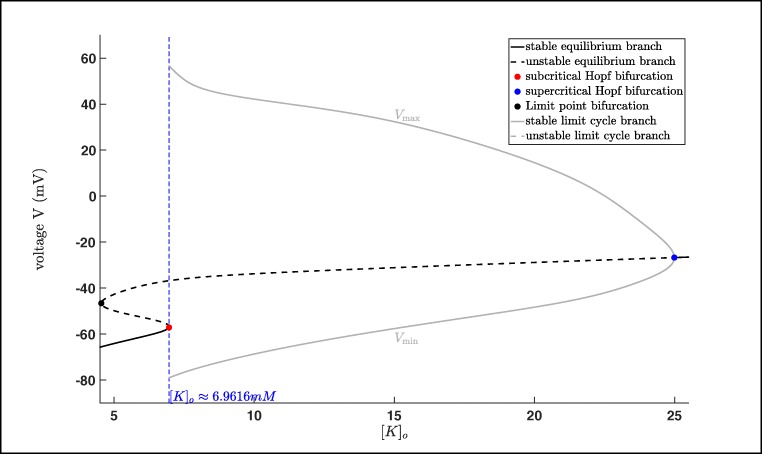
Potassium dynamics: Bifurcation diagram of the reduced system using [*K*]_*o*_ as bifurcation parameter (projection on the ([*K*]_*o*_,*V* )-plane)

In Fig. 10 we state the bifurcation diagram of the reduced system using [*K*]_*o*_ as bifurcation parameter to investigate the potassium dynamics of the system. Similar to Fig. 7 we have an equilibrium curve divided into two stable parts (black solid line) and one unstable part (black dashed line). The equilibrium curve loses stability via a subcritical Andronov–Hopf bifurcation (red dot, [*K*]_*o*_ ≈ 6.9616 *mM*), turns via a limit point bifurcation (black dot, [*K*]_*o*_ ≈ 4.5449 *mM*) and becomes stable again after the supercritical Andronov–Hopf bifurcation (blue dot, [*K*]_*o*_ ≈ 24.9893 *mM*). From the subcritical Andronov–Hopf bifurcation an unstable limit cycle branch bifurcates, which terminates at a homoclinic bifurcation, while from the supercritical Andronov–Hopf bifurcation a stable limit cycle branch bifurcates, which terminates at the subcritical Andronov–Hopf bifurcation. Here, we have to remark that trajectories of the reduced system will converge after certain amount of time (depending on the initial values) into either a stable equilibrium or a stable limit cycle depending on the value of [*K*]_*o*_. Furthermore, we know that [*K*]_*o*_ in the full system (1) depends on *K*_bath_ which is also reflected by comparing the trajectory of system (1) and the potassium dynamics of the reduced system.

**Fig. 11:**
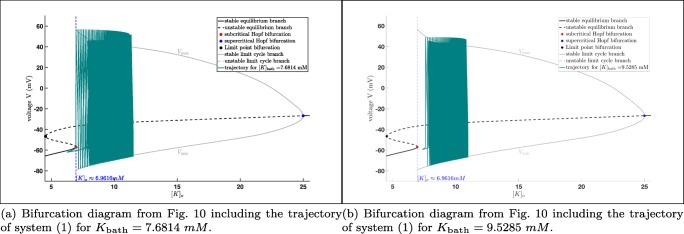
Bifurcation diagram of the reduced system using [*K*]_*o*_ as bifurcation parameter including seizures occurring in the full system (1) (projection on the ([*K*]_*o*_,*V* )-plane).

In Fig. 11, we have two examples of seizures produced by changing the value of *K*_bath_ (left and right ends of the *K*_bath_ interval – [7.6814;9.5285] – where seizures in system (1) appear). Here, we see that while seizures occur in system (1) the trajectories do not fit perfectly with the stable limit cycle branch of the reduced system, cf. Cymbalyuk and Shilnikov (2005) and Shilnikov (2012). Nevertheless, the trajectory is still attracted. Notice that the period *T* of the limit cycle corresponding to *K*_bath_ = 7.6814 *mM* is *T* ≈ 1026 *ms*, while the period of the limit cycle corresponding to *K*_bath_ = 9.5285 *mM* is *T* ≈ 42 *ms*. However, the trajectory corresponding to *K*_bath_ = 7.6814 *mM* has a spike train which lasts for approximately 600 *ms* and repeats every (approximately) 9.4 *s*, while the trajectory corresponding to *K*_bath_ = 9.5285 *mM* has a spike train which lasts for approximately 11.7 *s* and repeats every (approximately) 14.3 *s*. In addition, from Fig. 11 it is obvious that the maximal amplitude is decreasing for increasing values of *K*_bath_.

**Fig. 12:**
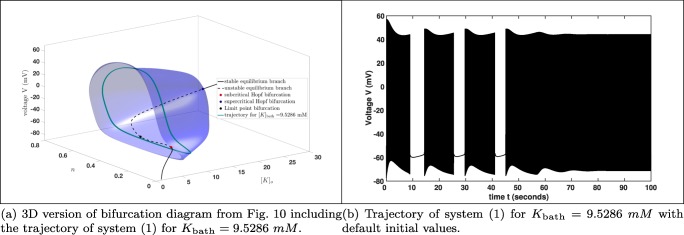
3D bifurcation diagram: Fig. 7 projection onto the ([*K*]_*o*_,*n*,*V* )-space including the trajectory for *K*_bath_ = 9.5286 *mM* after it converged into a stable limit cycle

Furthermore, we already saw in Fig. 9 that the trajectory converges into a stable limit cycle for *K*_bath_ large enough. The same effect we also have in Fig. 12, which means that the potassium dynamics of the reduced system coincides with the full system (1) provided *K*_bath_ is large enough. Notice that for values greater than *K*_bath_ = 9.529 *mM* the trajectory basically converges immediately into a stable limit cycle. Here, we saw the interplay between the potassium dynamics and the influence of the *K*_bath_. This indicates that on the one hand it makes sense to study a reduced system, on the other hand we saw also the loss of information, i.e. the torus bifurcation, does not appear in the reduced system. Beside this we will see in the next subsection that there are also other reasons for the occurrences of seizures, but the existences of a torus bifurcation indicates a region where seizures may appear. Notice also that using MATCONT, we can also show that the system, which is used for the bifurcation analysis in Wei et al. (2014b) exhibits also a torus bifurcation. Nevertheless, it is clear that the appearance of seizures is related to the extracellular potassium concentration [*K*]_*o*_ reflected in the potassium dynamics of system (1). But to derive more detailed results it is important to study the full system.

### Bifurcation analysis with respect to the amplitude of the ECT stimulus

Finally, on the cellular level, we investigate the effect of an ECT stimulus. To this end, we choose the amplitude *A* of Eq. (8) as bifurcation parameter.

**Fig. 13:**
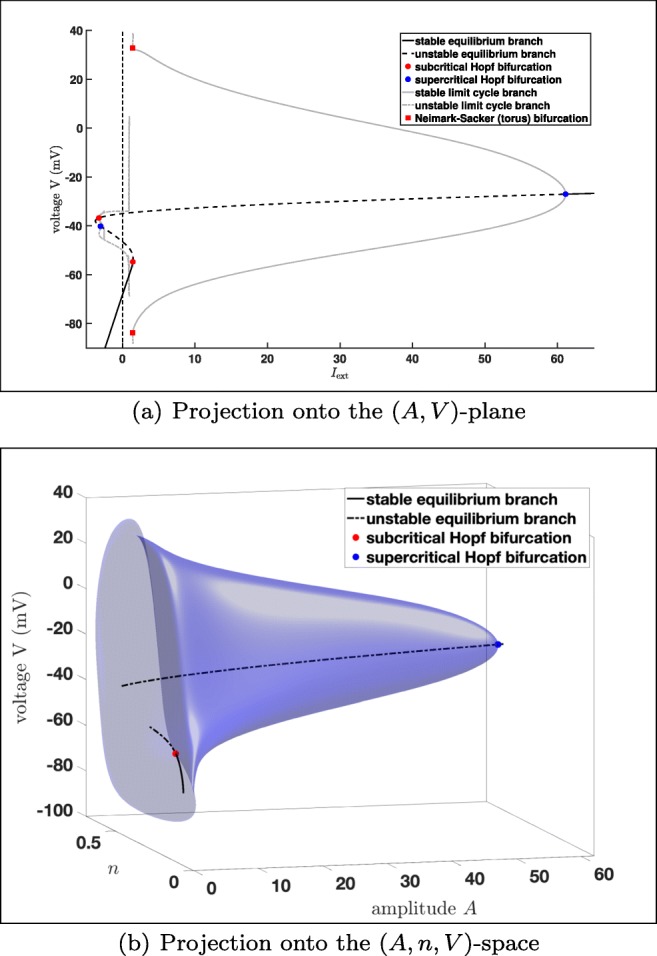
Bifurcation diagram of system (3) with respect to the amplitude *A*: **a** 2D bifurcation diagram of system (3): projection onto the (*A*,*V* )-plane. **b** 3D bifurcation diagram of system (3): Fig. 13 projection onto the (*A*,*n*,*V* )-space

The bifurcation diagram in Fig. 13 shows that the equilibrium curve is divided into two stable parts (black solid lines) and one unstable part (black dashed line). The equilibrium curve turns for a negatives amplitude *A*, which we did not include, since it is physiologically irrelevant. We have two Andronov–Hopf bifurcations, i.e. a subcritical one (red dot) and a supercritical one (blue dot). From the subcritical Andronov–Hopf bifurcation an unstable limit cycle branch bifurcates, which collides with the unstable equilibrium curve and disappears via a homoclinic bifurcation. While from the supercritical Andronov–Hopf bifurcation a stable limit cycle branch bifurcates, which becomes unstable via a torus bifurcation.

**Fig. 14:**
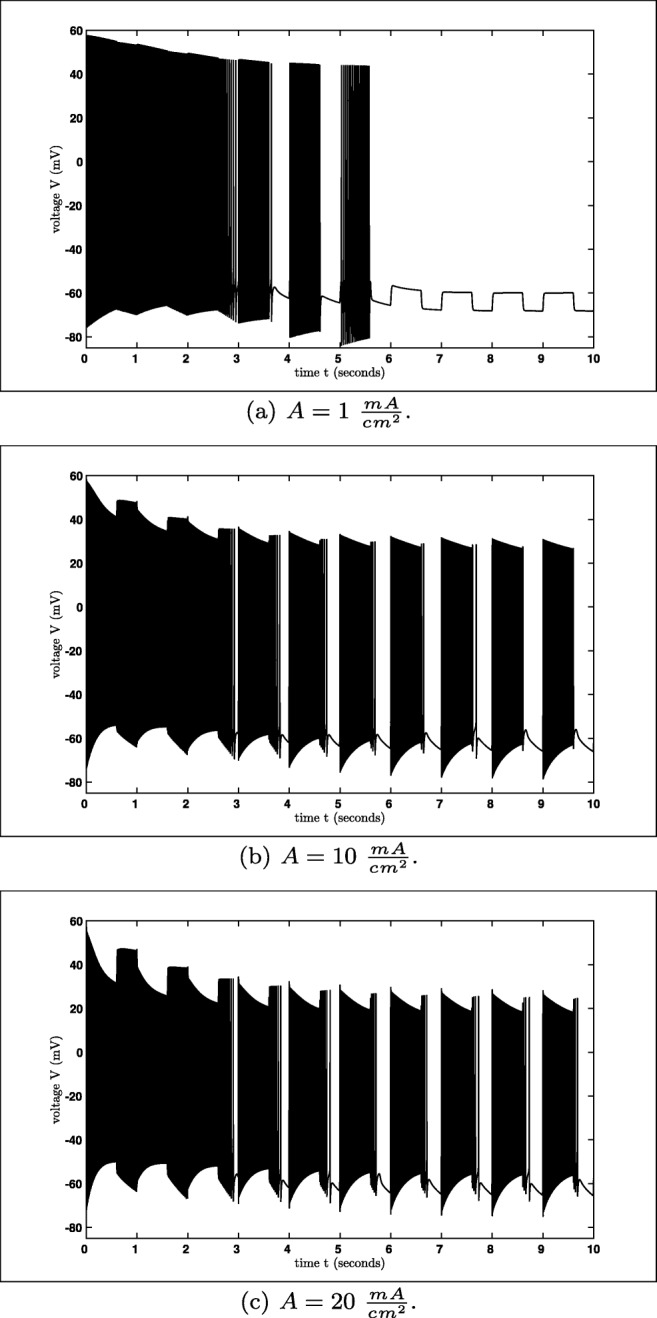
Simulation of the excited model for different amplitudes *A*

Notice that the situation here is different from the previous observations, since even if the system reaches a stable state the next external pulse will excite the system. Nevertheless, the bifurcation diagram will give an insight into the behaviour of the excited system. In Fig. 14 three stimuli with different amplitudes are simulated over 10 seconds. In general, the system needs (using the default initial values) certain amount of time to reach its ’stable’ behaviour. For $A=1\ \frac {mA}{cm^{2}}$ the system stops spiking after approximately 6 *s* – after this time only the stimulus is visible. Furthermore, one can see that the maximal amplitude of the spike trains is decreasing if the amplitude increases. Notice that the frequencies and the duration are always equal, since we have chosen the period *T* = 1000 *ms* and the duration *d* = 600 *ms*, but the number of spikes per spike train might be varying for different values of the amplitude *A*.

## Seizures on the macro–scale (*cm*)

The discussion has so far focused exclusively on the behaviour of a single cell. An extended model can carry the analysis over to the macro–scale, where many cells are connected together. The macro–scale is introduced to examine whether the potassium dynamics are still apparent when cells are coupled together. To this end, we introduce the monodomain model:

9 $$ \begin{array}{@{}rcl@{}} \frac{\lambda}{1+\lambda}\frac{1}{\chi}\nabla\cdot\left( M_{i}\nabla V\right) &=& C_{m}\frac{\partial V}{\partial t} + I_{\text{ion}},\\ \mathrm{n}\cdot\left( M_{i}\nabla V\right) &=& 0, \end{array} $$

where *M*_*i*_ denotes the intracellular conductivity tensor, *λ* the extra- to intracellular conductivity ratio and *χ* is the membrane surface area per unit volume, which is commonly used to model myocardial tissue Sundnes et al. (2006). Following Dougherty et al. (2014), we use *C*_*m*_ = 1 *μFcm*^2^, *χ* = 1260 *cm*^2^*cm*^3^, an extracellular conductivity of *M*_*e*_ = 2.76 *mScm* and an intracellular conductivity of *M*_*i*_ = 1 *mScm*. These conductivities give *λ* = *M*_*e*_*M*_*i*_ = 2.76. Notice that we have *I*_ion_ = (*I*_Cl_ + *I*_Na_ + *I*_K_), where the different ion currents are modelled by the system (1), i.e. we consider an ODE–PDE system coupling between system (1) and system (9). The system (9) is discretised in time with a second order Strang splitting scheme. The PDE is discretised in space with the finite element method. A time step of 0.025 *ms* is used and the characteristic mesh cell size is 0.002 *cm*. The solver is based on Rognes et al. (2017). The codes are available at https://github.com/jakobes/SeizureExperiments/tree/masterfor the monodomain model in 1D and 2D as well as for the bidomain model in 2D. Additionally, there are also movies provided for three main simulations, i.e. the spreading of action potentials in the monodomain and the bidomain model, to illustrate the behaviour of the macro–scale models.

**Fig. 15:**
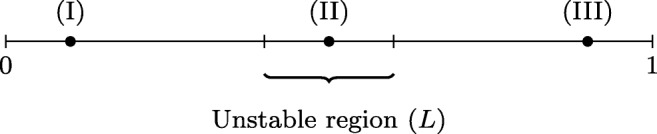
An illustration of the set up of our numerical experiment. The system (9) is solved on the entire domain [0,1] which measures 1 *cm*

The coupled system (9) is solved in the domain sketched in Fig. 15. The domain is split into two regions, the *unstable region* centred around the point (II), surrounded on either side by *stable regions*, (I) and (III). The instability will primarily be produced by changing the level of the potassium bath concentration, *K*_bath_, but also by applying an external stimulus. The behaviour of the system will be judged by sampling the computed transmembrane potential at eleven evenly spaced points along the domain, including (I), (II) and (III) from Fig. 15. The points (I) and (III) are positioned 0.1 *cm* from the end of the domain.

Two new parameters emerge with the introduction of the monodomain model, namely the intracellular conductivity *M*_*i*_, and, the length of the unstable domain *L*. We propose a set of experiments to examine the dynamics of the coupled system and how they change with *M*_*i*_ and *L*. We choose to concentrate on the extracellular potassium diffusion as it can be described by only *K*_bath_, rather than the external stimulus, which is described by three parameters, namely the amplitude, the duration of a square pulse and the frequency. In all the experiments, system (9) is solved in the domain described in Fig. 15, where the *K*_bath_ concentration is higher in the central unstable region, and lower in the surrounding regions.

**Fig. 16:**
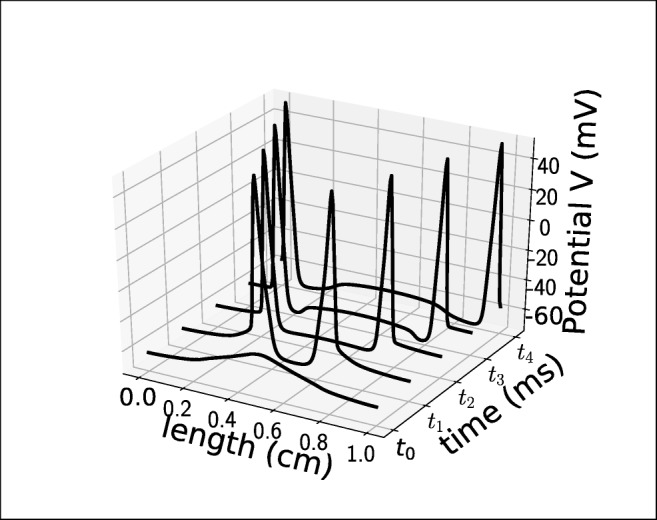
Five snapshots of an action potential spreading. The action potential is initiated soon after *t*_0_ = 975 *ms*, and vanishes out of the domain soon after *t*_4_ = 983 *ms*. This behaviour is representative of the behaviour of the coupled system (9) throughout all the experiments

In the following, let the value of *K*_bath_ in the central region be denoted by *K* bath*u* and *K* bath*s* in the surrounding region. The coupled system (9) is solved for 100 seconds, and we are interested in how the spatial coupling modifies the cell model behaviour with respect to bursting. In all the experiments, spikes are initiated in the central region and spread towards the edges of the domain. The manner in which the spikes spread is illustrated in Fig. 16.

**Fig. 17:**
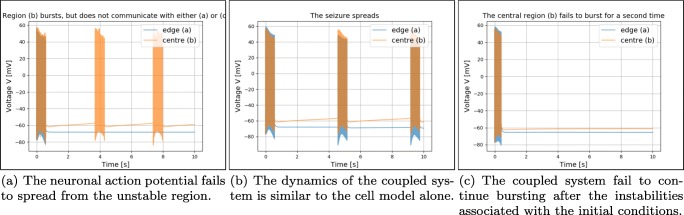
Three figures illustrating the three different kinds of modifications the monodomain model has on the cell model (1). The two lines are sampled at two different locations, cf. Fig. 15 , the unstable region (yellow line) measured at (II) and the stable region (blue line) measured at (I). When the blue and yellow lines overlap they turn brown

In each of the experiments, the simulation is carried out for a combination of conductivities and lengths of the unstable region. The conductivities are given by *M*_*i*_ = 2^*n*^*mScm* ,*n* = − 6,− 5,…,4, and the length of the unstable region is given by *L* = 2^*n*^*cm*,*n* = − 3,− 2,− 1. Three different kinds of behaviours emerge. Firstly, for conductivities typically close to *M*_*i*_ = 1 64 *mScm*, the unstable region will exhibit bursting behaviour, but the spikes will not spread to neighbouring cells. This is illustrated in Fig. 17a. Secondly, for medium range conductivities and for larger values of *L*, we observe the same behaviour in the coupled system as in the cell model alone, illustrated in Fig. 17b. Finally, for high conductivities, the stable region will dominate, and the unstable region will only produce a few initial spikes, and not burst again for the duration of the simulation, as seen in Fig. 17c. These spikes, however, will spread. These three kinds of behaviour of the system (9) will be referenced later in the discussion of the numerical experiments (Table 3).

**Table 3:** Table of values for *K*_bath_ used in experiment 1 through 5

Experiment #	*K* bath*s*	*K* bath*u*
1	4	8
2	6	8
3	4	9.5
4	4	10
5	2	8

Experiment 1 explores whether spikes will spread from the unstable region in Fig. 15 to the surrounding stable regions, where we choose *K* bath*s* and *K* bath*u* to produce two different regimes of system (1). *K*_bath_ = 4 *mM* is the default setting for the cellular model (1), while *K*_bath_ = 8 *mM* is the value used to study seizures. The number of spikes produced by system (9) in the first experiment is detailed in Table 4. One burst consists of about 200 spikes. The experiments in Tables 5, 6, 7 and 8 are all variations of this parameter configuration, exploring the effects of raising or lowering *K* bath*s* by 2 *mM*.

**Table 4:** Experiment 1, $K_{\text {bath}}^{s} = 4\ mM$, $K_{\text {bath}}^{u} = 8\ mM$

	Single Cell		Monodomain									
	*K*_bath_ = 4	*K*_bath_ = 8	1/64	1/32	1/16	1/8	1/4	1/2	1	2	4	8
0.12	5	675	236 - 679	675	674	670	449	198	133	31	9	7
0.25	5	675	247 - 679	675	675	674	671	554	203	147	68	17
0.5	5	675	239 - 679	675	675	674	674	671	669	211	170	126

**Table 5:** Experiment 2, $K_{\text {bath}}^{s} = 6\ mM$, $K_{\text {bath}}^{u} = 8\ mM$

	Single Cell		Monodomain									
	*K*_bath_ = 6	*K*_bath_ = 8	1/64	1/32	1/16	1/8	1/4	1/2	1	2	4	8
0.12	109	675	677	675	674	671	669	215	186	157	138	132
0.25	109	675	677	675	675	674	671	670	218	192	166	153
0.5	109	675	677	675	675	676	674	671	669	442	202	132

**Table 6:** Experiment 3, $K_{\text {bath}}^{s} = 4\ mM$, $K_{\text {bath}}^{u} = 9.5\ mM$

	Single Cell		Monodomain									
	*K*_bath_ = 4	*K*_bath_ = 9.5	1/64	1/32	1/16	1/8	1/4	1/2	1	2	4	8
0.12	5	1958	295 - 1956	1948	1938	1913	1772	1098	190	98	16	9
0.25	5	1958	295 - 1956	1955	1942	1937	1914	1780	1157	206	129	52
0.5	5	1958	296 - 1968	1959	1952	1945	1945	1780	1807	1370	432	180

**Table 7:** Experiment 4, $K_{\text {bath}}^{s} = 4\ mM$, $K_{\text {bath}}^{u} = 10\ mM$

	Single Cell		Monodomain									
	*K*_bath_ = 4	*K*_bath_ = 10	1/64	1/32	1/16	1/8	1/4	1/2	1	2	4	8
0.12	5	2891	308 - 2912	2889	2884	2862	2728	1401	207	113	21	10
0.25	5	2891	308 - 2913	2890	2888	2883	2862	2742	1621	224	143	69
0.5	5	2891	309 - 2914	2891	2890	2888	2884	2864	2770	1738	838	195

**Table 8:** Experiment 5, $K_{\text {bath}}^{s} = 2\ mM$, $K_{\text {bath}}^{u} = 8\ mM$

	Single Cell		Monodomain									
	*K*_bath_ = 2	*K*_bath_ = 8	1/64	1/32	1/16	1/8	1/4	1/2	1	2	4	8
0.12	2	675	201 - 715	223 - 277	674	672	449	180	54	5	3	3
0.25	2	675	201 - 731	225 - 678	675	674	672	450	188	88	7	4
0.5	2	675	204 - 727	228 - 678	675	675	674	673	451	200	131	34

In experiment 1, the first kind of behaviour is found only for *M*_*i*_ = 1 64 *mScm*, and the third is observed gradually from *M*_*i*_ = 1 4 *mScm*, but is offset by increasing *L*. These behaviours can be clearly seen, first by the huge range in the number of spikes seen in the first column in Table 4, and secondly by the decrease in the number of spikes in jumps of around 200 spikes starting at *M*_*i*_ = 1 4 *mScm*. Notably, in experiment 2, where *K* bath*s* is set to 6 *mM* detailed in Table 5, the first kind of behaviour of the system is no longer there for *M*_*i*_ = 1 64 *mScm*, and the transition to the third kind of behaviour starts for larger conductivities than in experiment 1, that is at *M*_*i*_ = 1 2 *mScm*. In the third experiment found in Table 6, *K* bath*u* is raised to 9.5 *mM*. The overall behaviour is similar to the first experiment in that only the first burst will spread out of the unstable region, and the remaining six will not. An obvious result of increasing the concentration of *K* bath*u* is that the system exhibits more bursts (seven in total) than experiment 1. However, the number of burst will still decline for *M*_*i*_ greater than 14 *mScm*, only more quickly than in the experiments 1 and 2. In the fifth experiment detailed in Table 8, the *K* bath*s* concentration is decreased by 2 *mM* compared to the first experiment. Interestingly, the first kind of behaviour is now also apparent for *M*_*i*_ = 1 32 *mScm*. Furthermore, from *M*_*i*_ ≥ 2*mScm*, the system will not get past the first burst for any of the tested values of *L*.

The final experiment, see Table 7, is different since *Kbathu* is chosen such that the ODE (1) converges into a stable limit cycle as discussed in Fig. 9. However, the time it takes before the solution settles into a pattern of continuous spiking is greatly affected by the conductivity in the coupled system and the length of the unstable domain. Similar to the other experiments, for *M*_*i*_ = 1 64 *mScm*, only the unstable region will exhibit the sustained spiking behaviour. Starting at *M*_*i*_ = 1 2 *mScm*, there is a transition from continuous spiking to discrete bursts. At *M*_*i*_ = 2*mScm* this is affecting all three lengths of the unstable domain. Despite the qualitative change in the cell mode behaviour, the influence of the spatial coupling is still the same. The behaviour of the system is still dictated by the cell model, and it will eventually settle into a pattern of continuous bursting, but the time before this happens is affected by the parameter configuration of *M*_*i*_ and *L*.

Finally, to return to the question of ECT, a similar experiment is performed where the central region in Fig. 15 is destabilised using an external current, as in Fig. 6, and *K*_bath_ is kept at 4 *mM*. A notable difference to the other class of experiments is that with an applied external stimulus, bursts will communicate to the surrounding stable region, even for low conductivities, see Table 9. At *M*_*i*_ = 1 *mScm*, the other end of the scale, the stimulated region will seize spiking. This is the same as observed in previous experiments, but the transition happens later.

**Table 9:** Stimulus with $A = 3\ \frac {mA}{cm^{2}}$, *d* = 600 *ms*, *T* = 1000 *ms*

	Single Cell		Monodomain									
	No stimulus	Stimulus	1/64	1/32	1/16	1/8	1/4	1/2	1	2	4	8
0.12	5	5115	5102	5100	5099	5091	5076	4889	1986	666	277	176
0.25	5	5115	4745	4538	4564	4283	4325	4675	4266	3867	2938	367
0.5	5	5115	5103	5100	5100	5100	5099	5094	5094	4980	4612	3760

### Monodomain model in a two–dimensional domain

**Fig. 18:**
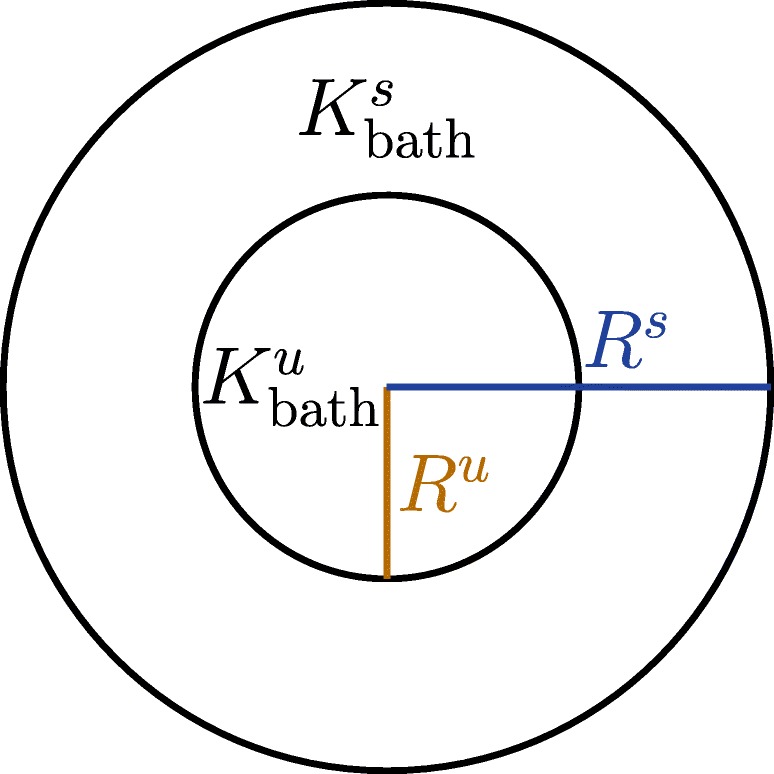
An illustration of two concentric circles where the innermost circle with radius *R*^*u*^ is the unstable region associated with $K_{\text {bath}}^{u}$. *R*^*u*^ is varied while the radius of the outermost circle, *R*^*s*^ is kept constant

In this section, we extend our investigation of the coupling of model (1) and the monodomain model (9) to a two–dimensional domain by considering two concentric circles as shown in Fig. 18.

A set of experiments similar to the 1D experiments, cf. Fig. 15 are performed. The region within the innermost circle is destabilised by the choice of $K^{u}_{\text {bath}} = 8\ mM$ or higher, the same way as in the 1D experiments in e.g. Table 4. The interaction between the size of the unstable region and the conductivity is again investigated. The simulations are limited to 10 seconds because 2D simulations are significantly more computationally demanding.

The size of the unstable region is characterised by the radius of the innermost circle. Three configurations of the circles are considered with the radius of the innermost circle, *R*^*u*^, set to *R*^*u*^ = 0.5 *cm*,0.25 *cm* and 0.125 *cm*. The radius of the outermost circle, *R*^*s*^, is kept fixed at 1 cm. A set of simulations with the same conductivities as in the 1D experiments are reported in Table 10.

**Table 10:** Experiment 7 — Concentric Circles with $K_{\text {bath}}^{s} = 4\ mM$ and $K_{\text {bath}}^{u} = 8\ mM$

	Single Cell		Monodomain									
	*K*_bath_ = 4	*K*_bath_ = 8	1/64	1/32	1/16	1/8	1/4	1/2	1	2	4	8
0.12	5	241	210	208	203	193	169	114	8	6	5	5
0.25	5	241	211	211	210	208	204	194	171	119	8	5
0.5	5	241	212	212	211	211	210	208	204	194	172	6

**Fig. 19:**
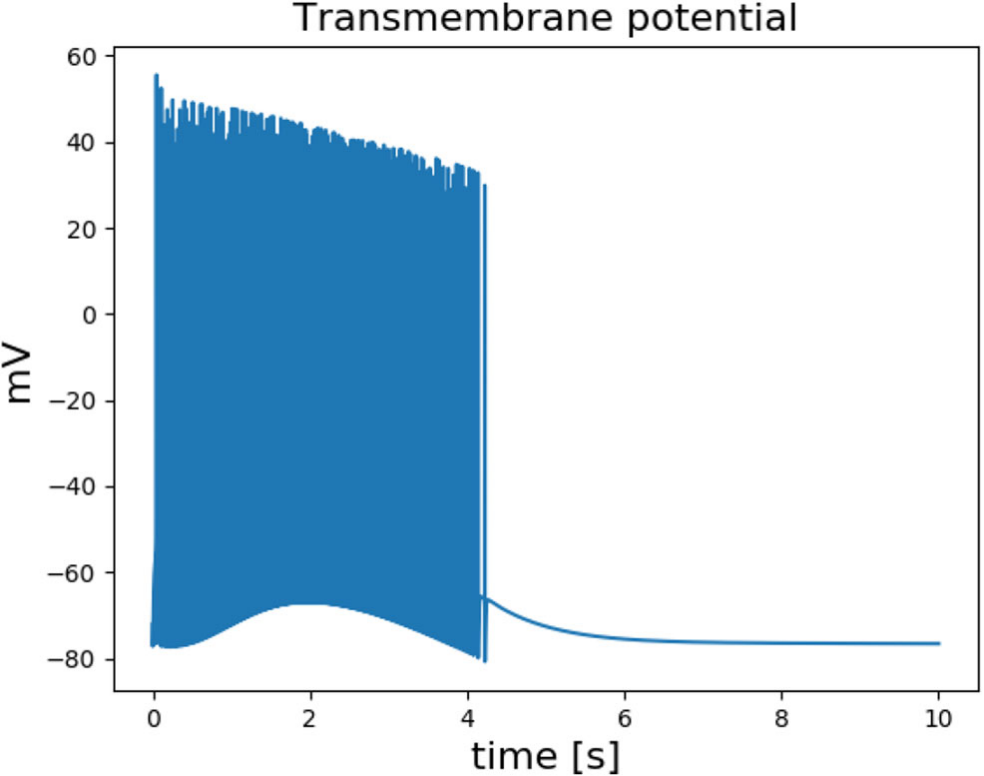
The transmembrane of the concentric circles 0.1 *cm* from the outermost circle

Action potentials are generated in the central unstable region and spread outwards, just as in the 1D simulations. The action potentials are grouped in bursts. During 10 seconds there is only one burst, see an example of the transmembrane potential in Fig. 19. The number of action potentials during the 10 seconds of simulation time is used to characterise the dynamics of the system.

The number of spikes in Table 10 for low conductivities is lower than in the cell model with *K*_bath_ = 8 *mM*. We remark that the number of spikes in the monodomain model is the same as or less than in the single cell model. However, there are a few instances where the number of spikes are slightly higher for the monodomain model, see Table 4. The experiment in Table 10 is similar to the 1D simulations in that the number of spikes increases with the size of the unstable region, and decreases with increased conductivity. The number of spikes decreases to the same level as the cell model with *K*_bath_ = 4 *mM*. These observations are confirmed by similar experiments performed for the remaining combinations of *K*^*s*^ and *K*^*u*^ listed in Table 3. These experiments can be found in the Supplementary Materials.

In Table 10 there is a sharp drop in the number of spikes in the rightmost part of the table where the number of spikes are below 10. The drop has a triangular shape suggesting that for smaller *R*^*u*^ similar drops will be present also for smaller conductivities. Similar drops were also found in Tables 4–9. We therefore hypothesised that the coupled system tends to some fixed small number of spikes as $R^{u} \rightarrow 0$ as seen in Table 10 for conductivities below a certain size. To test this hypothesis we extended the experiment in Table 10 with even smaller values of *R*^*u*^, $2^{-6}, 2^{-7} \dots , 2^{-10}$ for a fixed value of *M*_*i*_ = 1/16. With *R*^*u*^ = 2^− 6^ the system spiked 11 times and with *R*^*u*^ = 2^− 7^ to *R*^*u*^ = 2^− 10^ it spiked 9 times. This result indicates that for sufficiently small values of *R*^*u*^, the system will tend to a small number of spikes.

### Bidomain model in a two–dimensional domain

**Fig. 20:**
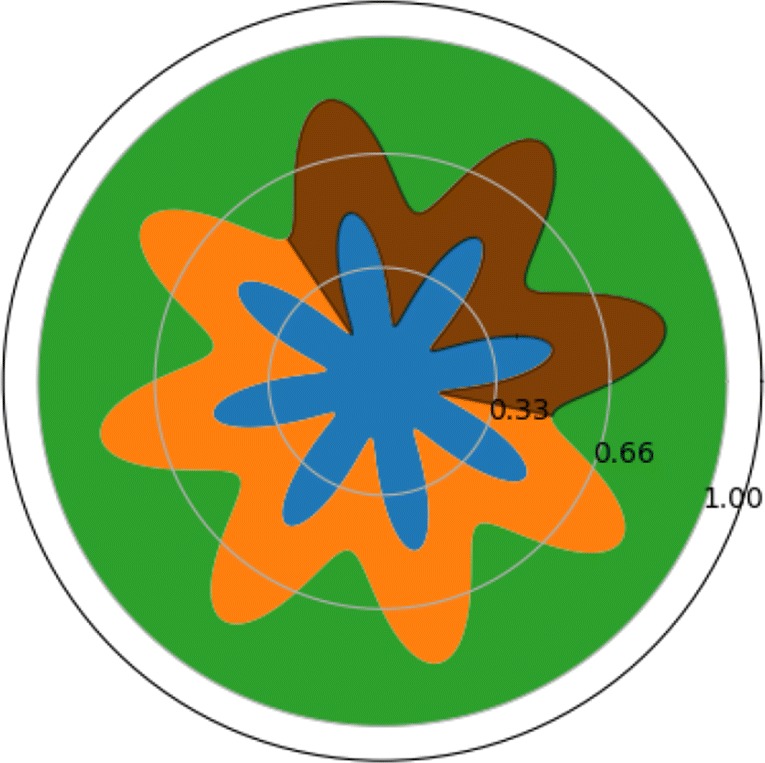
A rose geometry inspired by the fold in the human cortex. The green area is the cerebrospinal fluid, the blue are is the white matter. Both the orange and brown regions part of the grey matter, but the brown region is destabilised with *K*_bath_ = 8 *mM* and *K*_bath_ = 2 *mM*. The radius of the unstable region is 0.5 *cm* and $M_{i} = 1/64\frac {mS}{cm}$

The influence of the geometry is investigated by a variation of the concentric circles inspired by the folding of the human cortex. The domain is split into four distinct parts, green, orange, brown and blue, as seen in Fig. 20. The blue region represents the white matter, the orange and brown regions are the grey matter and the green part the cerebrospinal fluid (CSF). The geometry in Fig. 20 looks like a rose with eight petals. The unstable geometry varies from three petals, to two, to one petal. The number of petals in the unstable, marked in brown in Fig. 20 is denoted *P*^*u*^.

The behaviour of the monodomain model and the single cell model is quite similar in terms of the number of spikes, for physiologically relevant parameters. Here, we therefore extend the discussion also to the bidomain equations that handles anisotropic intracellular and extracellular media. Furthermore, we let model (1) be represented in the grey matter while the white matter is either passive or modelled by a simple neuronal model like Morris–Lecar (Gutkin and Ermentrout 1998; Tsumoto et al. 2006). In addition, we also include the CSF which bathes the brain. The equations are

10 $$ \begin{array}{@{}rcl@{}} C_{m} \frac{\partial V}{\partial t} -\frac{1}{\chi} \nabla \cdot (M_{i} \nabla (U_{e} + V)) &=&-I_{\text{ion}} ,\\ \nabla \cdot (M_{i}\nabla(U_{e} + V)) + \nabla\cdot(M_{e}\nabla U_{e}) &=& 0 \end{array} $$

for *x* ∈Ω, where Ω is the computational domain and equipped with the Neumann boundary condition

11 $$ \begin{array}{@{}rcl@{}} \mathrm{n}\cdot (M_{i} \nabla V + M_{i} \nabla U_{e}) &=& 0 \quad x \in \partial{\Omega}, \\ \mathrm{n}\cdot (M_{e} \nabla U_{e}) &=& 0\quad x \in \partial{\Omega}. \end{array} $$

The parameters are the same as in the monodomain model (9), except that the extracellular conductivity, *M*_*i*_ appears, and that *U*_*e*_, the extracellular potential appears as an unknown. The cerebrospinal fluid (CSF) is treated as part of the extracellular space with an intracellular conductivity of $10^{-12}\frac {mS}{cm}$. The extracellular conductivity, *M*_*e*_ is $1.26\frac {mS}{cm}$ in the white matter, and $2.76\frac {mS}{cm}$ in the grey matter. The white matter is modelled as passive, i.e. there is no cell model to govern cell dynamics there. The bidomain model is coupled to model (1) in the grey matter, but not in the CSF nor the white matter. The grey matter is mostly made up of cell bodies, while the white matter consists mostly of myelinated axons. There are ion channels in the axons, at the nodes of Ranvier, but it is not clear that model (1) is suitable.

An alternative, simple, cell model for the white matter is the Morris–Lecar model, (Gutkin and Ermentrout 1998; Tsumoto et al. 2006). It is a Hodking–Huxley model with two ion channels, sodium and potassium, unlike model (1). Numerical experiments similar to the ones in Table 11 showed increased oscillations in the intra- and extracellular potentials in the white matter, but oscillations were of insufficient amplitude to propagate the action potentials. The Morris–Lecar model with its default parameters is not suitable for modelling the propagation of action potentials in the white matter.

**Table 11:** Experiment 12 — Rose geometry with $K_{\text {bath}}^{s} = 4\ mM$ and $K_{\text {bath}}^{u} = 8\ mM$

	Single Cell		Bidomain									
	*K*_bath_ = 4	*K*_bath_ = 8	1/64	1/32	1/16	1/8	1/4	1/2	1	2	4	8
1	5	241	485	511	533	563	592	607	605	609	574	568
2	5	241	507	542	574	613	647	655	663	671	646	626
3	5	241	512	559	597	633	664	683	668	644	651	651

**Fig. 21:**
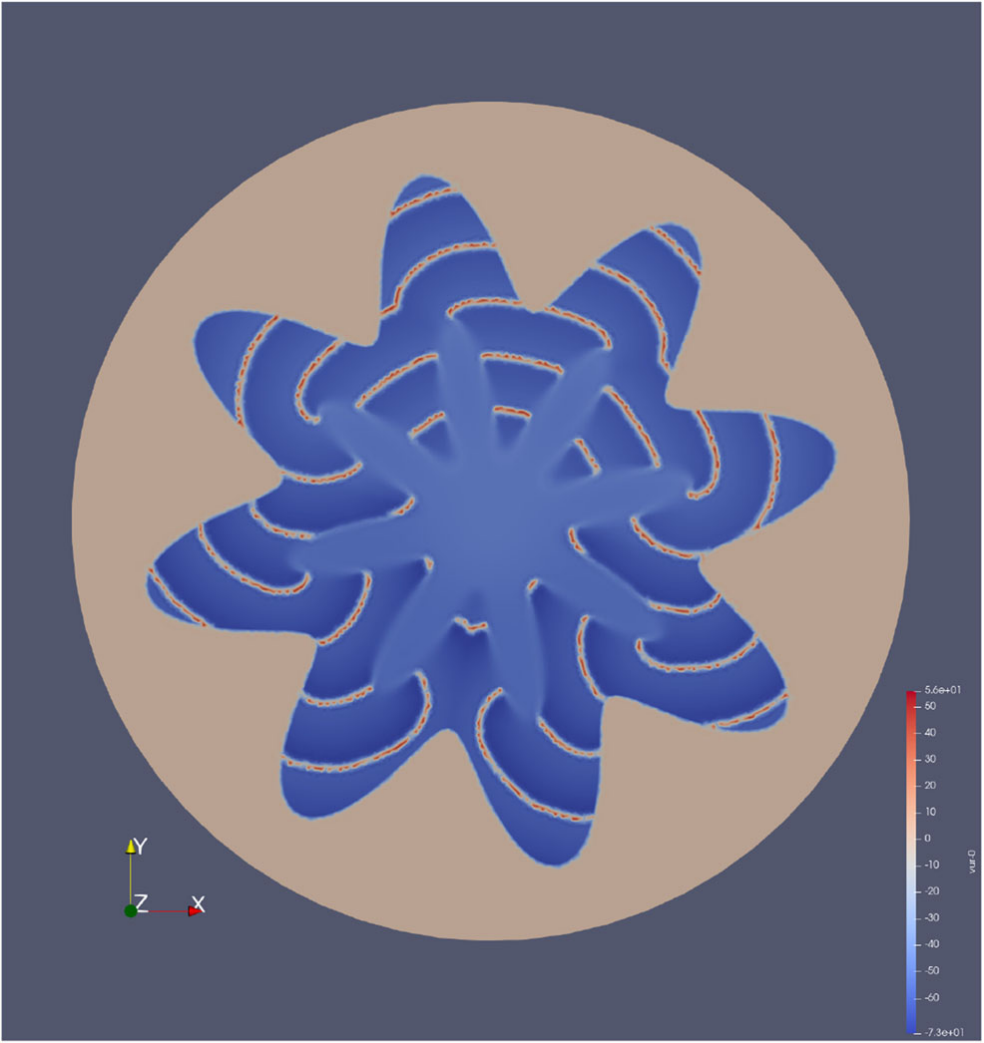
Waves of action potentials originating in the unstable region and following the outline of the grey matter. The unstable region encompasses three petals and $M_{i} = 1/64\frac {mS}{cm}$, $K_{\text {bath}}^{u} = 10\ mM$ amd $K_{\text {bath}}^{s} = 4\ mM$

Action potentials are generated in the unstable domain, close to the interface between the grey matter and the white matter. This is illustrated in Fig. 21.

The action potentials follow the outline of the grey matter until they meet. The set of experiments using the rose geometry follows the same pattern as the 1D experiments and experiments involving concentric circles. The number of action potentials is used to characterise the dynamics of the coupled system as the conductivity and the size of the unstable domain varies. The number of spikes increases with the size of the unstable region. This is the same as is observed in the experiments listed in Tables 4 and 10. In the rose experiment, the number of action potentials increases with the conductivity until $M_{i} = 1\frac {mS}{cm}$, after which it decreases again. This is in contrast to the experiments in Tables 4 and 10.

Another observation is that the coupled system has significantly more spikes than in the cell model. For very low intracellular conductivities the number of spikes approaches that of the cell model, but for higher intracellular conductivities, the number of spikes seems to stabilise at a level well above the cell model.

## Discussion

In this paper we investigated the ODE model (1) describing a neuron–glia cell interaction based on previous models from Barreto and Cressman (2011); Cressman et al. (2009, 2011). The major question, when does a normal action potential turn into seizures and which mechanism is behind that, is analysed and emphasised. Our main focus was the potassium dynamics, i.e. the influence of an enhancement or deficit of the extracellular potassium concentration [*K*]_*o*_ on the full system and mainly on the occurrence of seizures. To this end, our study is based on a suitable bifurcation analysis to derive a clear result how the system parameters have to be modified such that seizures appear. Here, we restricted ourself to the investigation of the influence of the potassium diffusion to the nearby reservoir *I*_diff_ and the potassium current *I*_K_.

Our study shows that an enhancement of the extracellular potassium concentration, which influences the Nernst potential of the potassium current, may lead to seizures, cf. Fig. 3 and Fig. 11. One reason is an enhancement in the potassium concentration nearby the reservoir *K*_bath_ and the existence of a torus bifurcation, cf. Section 2.1 and Fig. 7. A further reason is a deficit in the potassium leak current, cf. Supplementary File. Roughly speaking, we have shown that an increase in the extracellular potassium concentration [*K*]_*o*_ may yield seizure. This can be induced by an increase in the potassium concentration in the reservoir *K*_bath_ or a deficit in the potassium leak current *I*_KL_. Furthermore, one can assume that a similar effect will appear for corresponding enhancement or reduction in the currents *I*_pump_ and *I*_glia_.

The second main aim of this work is the study of the influence of ECT stimuli, which is used to induced seizures. Therefore, we introduced a suitable ODE system describing unidirectional rectangular pluses, which we coupled with system (1). This external forcing is affecting the system and has a big influence on the trajectories of system (1). Based on these investigations and the knowledge that ECT stimulus are used for the treatment of depressions by inducing seizures Peterchev et al. (2010), we modelled an ECT stimulus as a system of autonomous ODEs, cf. system (7) and Eq. (8), and then, we studied its influence on the cellular model (1). It turns out that – similar to the extracellular potassium concentration – the ECT stimulus may induce seizures, see Section 2.3. This autonomous ODE system then describes the ECT stimulus and it produces seizures in the neuron, i.e. we are coupling the cell model (1) with the ETC stimulus model. Also, in this case our bifurcation analysis gives an insight how to choose the amplitude of Eq. (8) to initiate a seizure in the cell model (1), cf. Section 2.3. In Fig. 13 we established the needed bifurcation diagram which we were using to produce seizures in model (1) (default setting) via the ETC stimulus, cf. Fig. 14.

One limiting factor in our study is the complexity of the cell model (1), but our approach can be extended to more complex model, cf. Øyehaug et al. (2012) and Y Ho and Truccolo (2016).

Finally, we have shown that the cell model extends to the monodomain model with the action potential then being fast traveling waves. Still, the number of spikes or bursts with the monodomain model is closely related to the number of spikes and burst in the cell model. In fact, for physiologically relevant conductivities the number of spikes and burst are very similar. For the bidomain model, including grey and white matter and in both a circular geometry and a more complex geometrical configuration with folding, the number of spikes are increased by a factor between two and three for physiological relevant conductivities. Hence, from the numerical experiments it seems that the bifurcation analysis of the cell model extends more or less directly to the PDE models, but this is hard to verify with current bifurcation analysis tools.

The model can be further improved either by the consideration of a bidomain model in a geometrically more realistic domain as in Dougherty et al. (2014), Mori (2015), and Lopez-Rincon et al. (2020), or a 3D–1D model in an explicit cell geometry, along the lines found in Ying and Henriquez (2007). Alternative models including both the connectome and dynamical models of normal conditions and seizures have been considered in Jirsa et al. (2014), Sanz-Leon et al. (2015), Jirsa et al. (2017), Breakspear (2017), Olmi et al. (2019), and Lopes et al. (2020). To what extent these models extends directly to the bidomain setting with sufficient detail has to our knowledge not been explored.

Moreover, an additional extension of the bidomain model is the consideration of (extracellular) ion diffusion. Ion diffusion is one key in the understanding of e.g. cortical spreading depression (CSD). The propagation speed of the ion concentration waves in CSD is slow, $1 - 10\frac {mm}{min}$, cf. Yao et al. (2011), while the phenomena considered in this paper have a characteristic time of milliseconds (spikes) and seconds (bursts) (fast electrical waves in $\frac {m}{s}$ during a seizure). Due to these different speeds, we did not include the ion diffusion. However, a corresponding bidomain model with ion diffusion would be an improvement, since ion diffusion plays an important role for the occurrence of certain phenomena.

In conclusion, we have quantified the length and the duration of a seizure related to different system parameters, i.e. *K*_bath_ and *G*_KL_, and found that the simulation of seizures are within plausible physiological regime similar to that which in clinical practice is required to last more than 20 − 30 *s* to be considered successful Frey et al. (2001) and Girish et al. (2003). Furthermore, with physiological reasonable conductivities and a wide range of *K*_bath_ and *G*_KL_ we have showed that seizures spread into an almost synchronous behaviour.

## Electronic supplementary material

Below is the link to the electronic supplementary material.

(MP4 3.68 MB)

(MP4 4.96 MB)

(MP4 10.0 MB)

(PDF 1.29 MB)
