# Supplementary material for: Dynamics of a neuron–glia system: the occurrence of seizures and the influence of electroconvulsive stimuli: A mathematical and numerical study
Source: J Comput Neurosci. 2020 May 12;48(2):229–51. doi: 10.1007/s10827-020-00746-5 (PMC7242278; doi:10.1007/s10827-020-00746-5)
Supplement: Supplementary file 4 — (PDF 1.29 MB) [file 10827_2020_746_MOESM4_ESM.pdf]

myjournal manuscript No.
(will be inserted by the editor)

Supplementary file to the paper "Dynamics of a neuron–glia system: the occurrence of seizures and the influence of electroconvulsive stimuli"

A mathematical and numerical study

André H. Erhardt · Kent-Andre Mardal · Jakob E. Schreiner

the date of receipt and acceptance should be inserted later

1 Bifurcation analysis with respect to potassium leak current

Up to this stage we saw that the extracellular potassium concentration $[K]_o$ has an influence on the occurrence of seizures. Next, we consider the potassium leak current $I_{\text{KL}} = G_{\text{KL}}(V - E_K)$, which is part of the potassium current I_{K}, and its effect on the appearance of seizures to validate our observations from the previous section. In Fig. 1 two examples of seizures for a reduced potassium leak current are shown. Such behaviour is to expect regarding model (1) and our previous discussions in Section 2.1 and 2.2. Notice that the trajectory corresponding to $G_{\text{KL}} = 0.004755\ mM$ has a spike train which lasts for approximately 60 s and repeats every (approximately) 90 s, while the trajectory corresponding to $G_{\text{KL}} = 0.0081\ mM$ has a spike train which lasts for approximately 18.9 s and repeats every (approximately) 88 s. Here, we clearly see that a reduction of the potassium leak current or the potassium leak current conductance G_{KL}, respectively, may yield seizures. Moreover, notice that we have in this case a prolongation of the time interval compared to Fig. 2. In the following, we will discuss the influence of a deficit in the potassium leak current using G_{KL} as bifurcation parameter. Furthermore, we want to highlight that one can expect similar effect regarding a variation in I_{glia} or combinations of these currents. Again, we start deriving the equilibrium curve in this situation with respect to G_{KL}. Here, we have some slightly different mechanism leading to seizure. First of all, we have to highlight that there are two subcritical Andronov–Hopf bifurcations. Between these Andronov–Hopf bifurcations the equilibrium curve is unstable. Notice that one of these Andronov–Hopf bifurcations appears for a positive value, i.e. $G_{\text{KL}} \approx 0.008134\ \frac{mS}{cm^2}$, while the other one appears for a negative value, i.e. $G_{\text{KL}} \approx -4.745544\ \frac{mS}{cm^2}$. Therefore, usually one would ignore the negative part of the G_{KL}–axis in Fig. 2. But the limit cycle branch

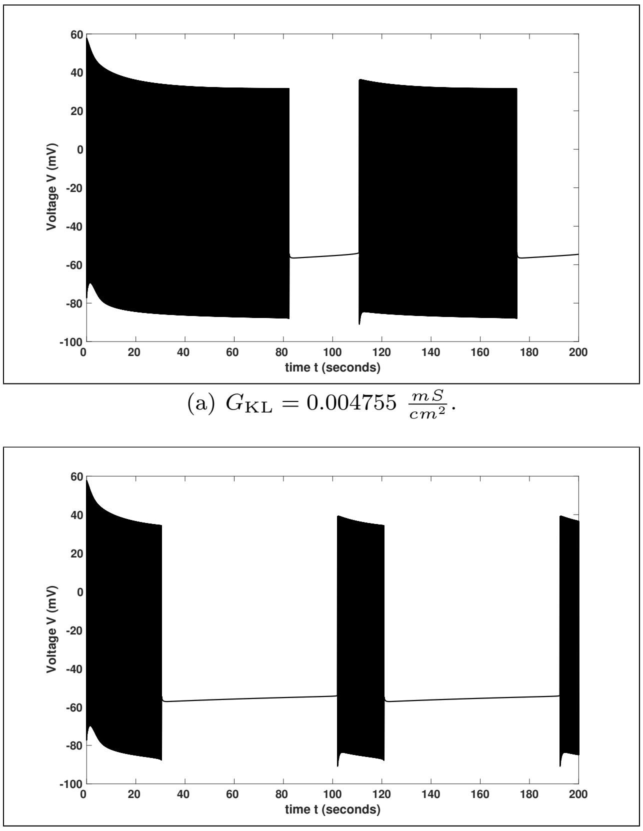

(a) $G_{\text{KL}} = 0.004755\ \frac{mS}{cm^2}$.

(b) $G_{\text{KL}} = 0.0081\ \frac{mS}{cm^2}$.

Fig. 1 Two examples of seizures appearing in system (1) for a reduced value of the potassium leak current conductance G_{KL}.

Address(es) of author(s) should be given

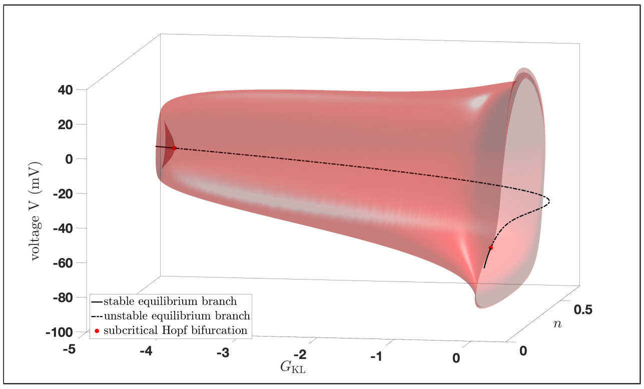

Fig. 2 Bifurcation diagram with respect to the potassium leak current conductance G_{KL}.

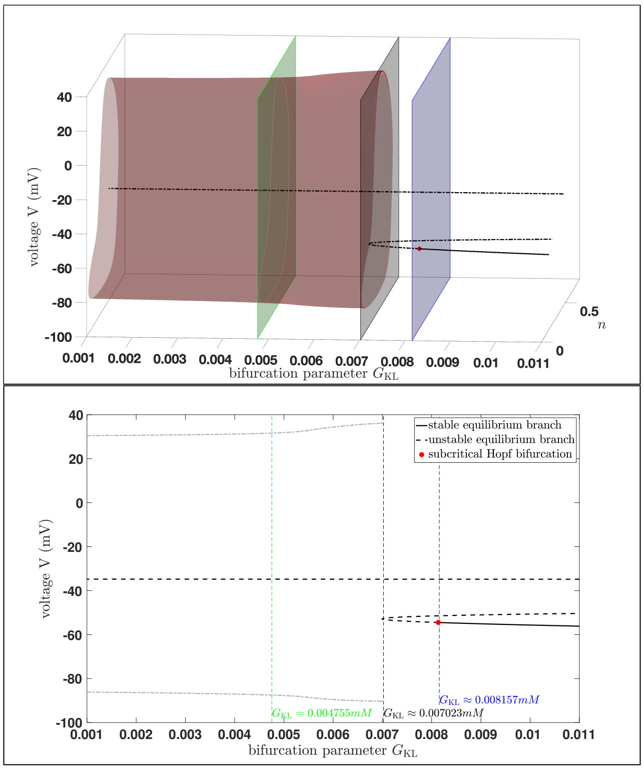

Fig. 3 Zoom of Fig. 2 as two and three dimensional bifurcation diagram.

which is bifurcating from this Andronov–Hopf bifurcation crosses from the negative part to the positive part of the G_{KL}–axis. Therefore, the limit cycle branch on the positive part of G_{KL}–axis affects the trajectory of system (1) in a physiological relevant region. Indeed, this limit cycle branch induces the seizures occurring with respect to G_{KL}. In Fig. 3 we zoom into the region, where seizures related to a deficit of the potassium leak current appear as a two and three dimensional projection. Here, we included three vertical lines, i.e. where the limit cycle branch collides with the unstable equilibrium curve (vertical black dashed line) and the left and right bound of the 'seizure region' (vertical green and blue dashed line). In this situation seizures occur not via a torus bifurcation. Nevertheless, we see in Fig. 3 that similar to Section 2.1 the unstable limit cycle branch 'opens up' and this region induces seizures.

2 2D simulations for several K_{bath} concentrations

The experiments in Tables 1, 2 and 3 all have $K^u_{\mathrm{bath}} = 8\ mM$ but with $K^s_{\mathrm{bath}} = 2, 4, 6\ mM$. The effect of increasing K^s_{bath} is seen for the highest values of the intracellular conductivity, as the lowest number of spikes grows from 2, to 5, to 89. Model (1) with $K_{\mathrm{bath}} = 2\ mM$ and $4\ mM$ will spike 2 and 5 times, respectively. Model (1) with $K_{\mathrm{bath}} = 6\ mM$ will spike 109 times, compared to the 89 in the concentric circle simulation. This is unlike the 1D experiment where for $M_i = 8\frac{mS}{cm}$ the spike count was still greater then in the cell model.

The number of action potentials does not vary for $M_i = \frac{1}{64}\frac{mS}{cm}$, the lowest conductivity considered here, this holds true for all the sizes of the unstable domain in these three experiments, and seem to be dictated by K^u_{bath}. The number of spikes in the cell model during 10 seconds with $K_{\mathrm{bath}} = 8\ mM$ is 241. The number of spikes drops off, forming an almost constant relation between R^u and M_i as the conductivity increases. There are, for example in Table 1, 211 action potentials for $(R^u, M_i) = (0.25, 1/64)$ and $(0.5, 1/16)$ respectively, and 210 action potentials for $(R^u, M_i) = (0.12, 1/64)$, $(0.25, 1/16)$ and $(0.5, 1/4)$. Similar patterns exist for higher conductivities in Table 1 and inTables 2, 3, 4 and 5. Common among all the experiments is that the effect of increasing K^s_{bath} serves to increase the number of action potentials for $M_i > \frac{1}{64}\frac{mS}{cm}$.

The experiments in Tables 3 and 4 investigate the effect of increasing K^u_{bath} compared to the experiment in Table 2. The effect is to increase the number of action potentials during the 10 seconds of simulation, except for $M_i = 4\frac{mS}{cm}$, where there are 5 action potentials in all three experiments. The constant relationships on the diagonals are preserved also with an increased K^u_{bath}.

Table 1 Experiment 6 — Concentric Circles with $K^s_{\mathrm{bath}} = 2\ mM$ and $K^u_{\mathrm{bath}} = 8\ mM$.

R^u \ M_i	1/64	1/16	1/4	1	4
0.12	210	202	148	4	2
0.25	211	210	202	151	4
0.5	212	211	210	203	153

Table 2 Experiment 7 — Concentric Circles with $K^s_{\text{bath}} = 4\ mM$ and $K^u_{\text{bath}} = 8\ mM$.

R^u \ M_i	1/64	1/16	1/4	1	4
0.12	210	203	169	8	5
0.25	211	210	204	171	8
0.5	212	211	210	204	172

Table 3 Experiment 8 — Concentric Circles with $K^s_{\text{bath}} = 6\ mM$ and $K^u_{\text{bath}} = 8\ mM$.

R^u \ M_i	1/64	1/16	1/4	1	4
0.12	210	205	185	143	89
0.25	211	210	205	186	144
0.5	212	211	210	205	187

Table 4 Experiment 9 — Concentric Circles with $K^s_{\text{bath}} = 4\ mM$ and $K^u_{\text{bath}} = 9.5\ mM$.

R^u \ M_i	1/64	1/16	1/4	1	4
0.12	262	255	218	84	5
0.25	263	262	255	220	95
0.5	264	263	262	255	221

Table 5 Experiment 10 — Concentric Circles with $K^s_{\text{bath}} = 4\ mM$ and $K^u_{\text{bath}} = 10\ mM$.

R^u \ M_i	1/64	1/16	1/4	1	4
0.12	280	272	232	113	5
0.25	282	280	272	234	118
0.5	282	282	280	273	235

2.1 The rose geometry

The effect of increasing K^s_{bath} is not so clear as it was in the experiments with the monodomain model and the concentric circles. In the rose experiments, the number of spikes is increased regardless of the size of the unstable domain or the conductivity. The same is true when K^u_{bath} is increased.

Table 6 Experiment 11 — Rose geometry with $K^s_{\text{bath}} = 2\ mM$ and $K^u_{\text{bath}} = 8\ mM$.

P^u \ M_i	1/64	1/16	1/4	1	4
1	470	507	555	563	550
2	504	567	634	645	627
3	511	594	659	685	641

Table 7 Experiment 12 — Rose geometry with $K^s_{\text{bath}} = 4\ mM$ and $K^u_{\text{bath}} = 8\ mM$.

P^u \ M_i	1/64	1/16	1/4	1	4
1	485	533	592	605	574
2	507	574	647	663	646
3	512	597	664	668	651

Table 8 Experiment 13 — Rose geometry with $K^s_{\text{bath}} = 6\ mM$ and $K^u_{\text{bath}} = 8\ mM$.

P^u \ M_i	1/64	1/16	1/4	1	4
1	499	561	632	652	626
2	510	582	661	682	670
3	513	600	669	691	663

Table 9 Experiment 14 — Rose geometry with $K^s_{\text{bath}} = 4\ mM$ and $K^u_{\text{bath}} = 9.5\ mM$.

P^u \ M_i	1/64	1/16	1/4	1	4
1	525	565	620	630	615
2	559	628	705	719	694
3	567	658	733	757	711

Table 10 Experiment 15 — Rose geometry with $K^s_{\text{bath}} = 4\ mM$ and $K^u_{\text{bath}} = 10\ mM$.

P^u \ M_i	1/64	1/16	1/4	1	4
1	538	576	630	639	622
2	578	648	727	740	712
3	587	681	760	785	734
